# Mammary and respiratory infection of sheep with H5Nx clade 2.3.4.4b viruses with milk-mediated transmission to lambs

**DOI:** 10.1126/sciadv.aed1287

**Published:** 2026-05-08

**Authors:** Tamiru N. Alkie, Carissa Embury-Hyatt, Anthony V. Signore, Estella Moffat, Darwin Ramos, Sugandha Raj, Tamiko Hisanaga, Ifeoluwa Ayilara, Daniel S. Sullivan, Patrick Lloyd, Lemarie Pama, Cassidy N. G. Erdelyan, Andrew S. Lang, Darwyn Kobasa, Davor Ojkic, Loren Matheson, Oliver Lung, Zvonimir Poljak, Yohannes Berhane

**Affiliations:** ^1^ Canadian Food Inspection Agency, National Centre for Foreign Animal Disease, Winnipeg, Manitoba, Canada.; ^2^ Department of Biology, Memorial University of Newfoundland, St. John’s, Canada.; ^3^ Special Pathogens Program, National Microbiology Laboratory, Public Health Agency of Canada, Winnipeg, Manitoba, Canada.; ^4^ Animal Health Laboratory, University of Guelph, Guelph, Canada.; ^5^ Centre for Security Science, Defence Research and Development Canada, Ottawa, Ontario, Canada.; ^6^ Department of Biological Sciences, University of Manitoba, Winnipeg, Manitoba, Canada.; ^7^ Department of Population Medicine, Ontario Veterinary College, University of Guelph, Guelph, Ontario, Canada.; ^8^ Department of Veterinary Pathology, Western College of Veterinary Medicine, University of Saskatchewan, Saskatoon, Canada.; ^9^ Department of Animal Science, University of Manitoba, Winnipeg, Manitoba, Canada.; ^10^ Department of Pathobiology, University of Guelph, Guelph, Ontario, Canada.

## Abstract

H5Nx clade 2.3.4.4b viruses are evolving rapidly, expanding host ranges and threatening animal and public health. In the US, genotype B3.13 dominates dairy outbreaks, while D1.1 is linked to fewer cases. In the UK, an asymptomatic ewe infected with genotype DI.2 raised concerns about ruminant susceptibility. We inoculated lactating and nonlactating sheep with D1.1 (H5N1) and A6 (H5N5) viruses. Intramammary inoculation in lactating sheep caused clinical mastitis, high viral loads in milk, and transmission to suckling lambs, which further spread infection to the uninoculated mammary glands. Both ewes and their lambs seroconverted. Aerosol exposure of nonlactating sheep led to transient respiratory infection, with low-level viral replication, and seroconversion. In vitro, both viruses replicated in sheep mammary epithelial cells. These findings establish sheep as a viable ruminant model for H5N1 and H5N5 infection and highlight previously unidentified transmission dynamics, including milk-mediated and lamb-to-ewe spread, relevant for surveillance and biosecurity in ruminant populations.

## Teaser

In nonlactating sheep, aerosolized H5Nx caused transient respiratory infection, while intramammary inoculation caused mastitis.

## INTRODUCTION

Since their emergence in 2021, H5N1 clade 2.3.4.4b viruses have undergone extensive genetic reassortments with endemic low-pathogenic avian influenza viruses, resulting in marked diversification of circulating H5N1 genotypes (*1*–*3*). In the Americas, H5N1 clade 2.3.4.4b genotypes B3.2 and B3.6 have affected a wide range of avian and mammalian hosts during their periods of peak incidence, persisted across multiple seasons (*1*, *2*, *4*), and particularly genotype B3.2 spread as far as the Antarctic regions (*5*, *6*). Phylogenetic clustering analysis indicates that the H5N1 genotype B3.6 represents the ancestral lineage for the H5N1 genotype B3.13 virus. Typically, these genotypes retained Eurasian hemagglutinin (HA; H5) and neuraminidase (N1) surface proteins (*1*). Conversely, the H5N1 clade 2.3.4.4b genotype D1.1 reported in late 2024 in the US and Western Canada carries a Eurasian-derived H5 and a North American–derived N1 surface protein (*7*, *8*), with certain virus isolates from domestic poultry outbreaks in Canada accumulating an antiviral resistance mutation in their NA protein (*8*). The panzootic H5N1 clade 2.3.4.4b viruses have a long NA stalk, providing these viruses with a broader host range and increased fitness in mammals (*9*). The H5N5 clade 2.3.4.4b genotype A6 has predominantly affected seabirds, as well as carnivorous birds and mammals, in Eastern Canada, Europe, and Russia since 2023 (*10*–*13*) and is characterized by the presence of a truncated NA stalk region (*11*), a recognized virulence marker in domestic poultry (*14*). Nevertheless, recent experimental study in ferrets and data from naturally infected mesocarnivorous mammals demonstrate that infection with the H5N5 genotype A6 is associated with high pathogenicity, severe neurologic disease, and rapid fatality (*11*).

The H5N1 clade 2.3.4.4b genotype B3.13 since detection in a dairy farm in Texas in 2024 has rapidly spread through 1074 herds of dairy cattle across 17 states (*15*) with spillover infections into domestic cats, mesocarnivores, and various avian species (*4*, *16*, *17*). Predictive modeling revealed higher infection rates in dairy cows associated with the genotype B3.13 than reported (*18*). In February 2025, the emerging H5N1 genotype D1.1 was confirmed in dairy cows across three states in the US (*19*), following its prior impact on diverse avian species, including domestic poultry (*7*, *8*). These outbreaks were limited to a small number of dairy herds because of swift restriction in cattle movements resulting from rapid confirmation by comprehensive bulk milk testing. From April 2024 to May 2025, of the 71 human cases reported in the US, 41 were related to exposure to H5N1-infected dairy cattle (*20*). Multiple introductions into dairy cattle are now hypothesized, as both genotypes B3.13 and D1.1 have been detected in infected cows (*16*, *17*, *21*). Despite clade 2.3.4.4b H5N1 having attained a panzootic status and the fact that multiple viral genotypes are circulating in birds (*4*), highly pathogenic avian influenza (HPAI) cases have never been confirmed in dairy cows in other countries (*22*–*26*). Two instances of HPAI infection have been reported in noncattle ruminants, one in kid goats caused by the genotype B3.6 in the US (*16*) and a second one in a lactating sheep in the UK caused by the genotype DI.2, which show the broad host range capabilities of the virus (*27*). Both cases were epidemiologically linked to infected birds. The latter case of H5N1 infection in a lactating ewe is a milestone in the emergence of H5N1 as a mammary pathogen in nonruminant livestock (*28*). The extent of H5N1 incidence in sheep and goats is unclear. Spillover of H5 subtype infection based on serologic evidence was reported in sheep and goats in Pakistan (*29*) and sheep in Norway (*30*), which coincided with the peak activity of clade 2.3.4.4b H5 in wild birds and domestic poultry.

The outcomes of natural infection with the genotype B3.13 in dairy cows are correlated with the stages and number of lactations as shown in a recent epidemiologic study on a large dairy farm in Ohio, US (*31*). Clinical influenza was more frequent in older, multiparous dairy cows in the late lactation stage compared to cows at the early lactation stage (*31*). Older and late-stage lactating Holstein cows exhibited more severe clinical disease with B3.13 mammary inoculation (*32*) compared to midstage lactating Holstein cows (*33*). Similar outcomes to cow experiments were observed when late-stage lactating goats were infected by combined respiratory and intramammary routes with the genotype B3.13 or B1.2 (*34*). Naturally or experimentally infected dairy cows developed clinical mastitis because of virus replication in the mammary glands (*16*, *32*, *33*, *35*) and shed substantial amounts of viruses in milk (*16*). Mammary glands in bovine species express both human-type (SAα-2,6 Gal) and avian-type (SAα-2,3Gal) sialic acid (SA) receptors (*36*–*39*). The mammary tropism of clade 2.3.4.4b viruses is associated with the mammary expression of the avian-type SA receptor in dairy cows (*36*, *37*). Analytics including HA sequencing, structural evaluations, and in vitro assays have shown currently circulating clade 2.3.4.4b H5Nx viruses, including the genotype B3.13, which have a preference for binding to the avian-type SA receptor (*40*–*42*). Respiratory inoculation in Holstein heifers and calves caused mild respiratory infection with low-level transient virus shedding (*32*, *33*). Human infections through oral exposure to raw milk from cows infected with B3.13 are yet unknown. However, in nonhuman primates, experimental oral exposure caused asymptomatic infection (*43*). All human cases related to the B3.13 virus from dairy sources appear linked to accidental conjunctival exposure (*44*, *45*).

Genotypes B3.13 and D1.1 were each introduced to dairy cows as a result of different spillover events from largely unidentified avian reservoir hosts (*17*, *46*). Unsampled poultry populations were also hypothesized as alternate sources for the genotype B3.13 (*17*). Interstate movements of infected dairy cows possibly enhanced successive viral spread at a larger scale between dairy herds (*16*, *17*). This is supported by the fact that transmissions of B3.13 continued, even though its prevalence in wild birds was declining (*17*). In this instance, infected cows could serve as reservoir hosts for spillback infections to wild birds or peridomestic animals, likely to have occurred while frequently visiting accumulated wastewater from dairy farms (*47*). The survival of the virus for prolonged periods on milking equipment presents substantial risks for mammary infections (*48*), although a recent experimental study failed to provide ample evidence supporting the hypothesis that milking equipment directly mediates infection of the mammary tissues in dairy cows (*49*). The routes by which dairy cows and lactating sheep acquire H5N1 infections and developed clinical mastitis remain poorly understood. Detection of high virus loads in the milk and on milking equipment plausibly suggests retrograde infections via the teat canal or transmission through mouth-to-teat contact. The emergence of genetically and antigenically diverse clade 2.3.4.4b viruses that affect ruminant livestock is likely to increase as these viruses continue to evolve in wild bird reservoirs. Given the emerging evidence of H5N1 infections in small ruminants and the lack of controlled pathogenesis studies, we investigated the susceptibility and transmission dynamics of H5Nx viruses in sheep as a representative ruminant model and specifically evaluated whether suckling lambs could serve as a conduit for virus transmission between the mammary glands. We found that nonlactating sheep infected through the respiratory route exhibited subclinical to mild infection, characterized by transient virus shedding from the upper respiratory system. In lactating sheep, intramammary inoculation of a single mammary gland induced clinical mastitis in the infected gland, with subsequent transmission detected in the contralateral, initially uninfected gland, likely facilitated by the suckling lambs.

## RESULTS

### Pathogenicity of Mink-H5N1 (D1.1) and Gull-H5N5 (A6) in nonlactating sheep

**Fig. 1.**
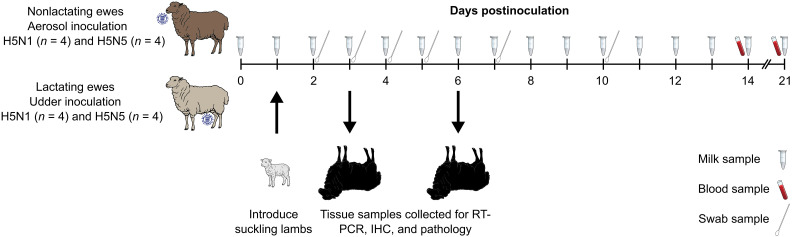
Respiratory (aerosol) and intramammary infections in sheep. Two groups of nonlactating sheep (*n* = 4 per virus) were inoculated with 10^6^ TCID_50_ of A/GBBG/NL/OTH0021-23/2025 (genotype A6, Gull-H5N5) or A/Mink/ON/FAV0083/2025 (genotype D1.1, Mink-H5N1) by a nebulizer (respiratory infection). Two groups of lactating sheep (*n* = 4 per virus) were intramammarily inoculated with 10^5^ TCID_50_ of genotype D1.1 or A6 virus via the teat canal into the right mammary gland. The left mammary gland was uninoculated to assess virus transmission by suckling lambs that were reunited with their mothers on 1 DPI. One sheep each was euthanized at 3 and 6 DPI from both infection groups. Sheep were monitored for clinical signs and rectal temperature; nasal and oral swabs and milk samples were collected at defined time points. Blood samples were collected at different time points. Images adapted from works by J. Li (https://scidraw.io/drawing/537) and D. De Oliveira (https://scidraw.io/drawing/528) under creative commons license (CC BY 4.0; https://creativecommons.org/licenses/by/4.0/deed.en).

**Fig. 2.**
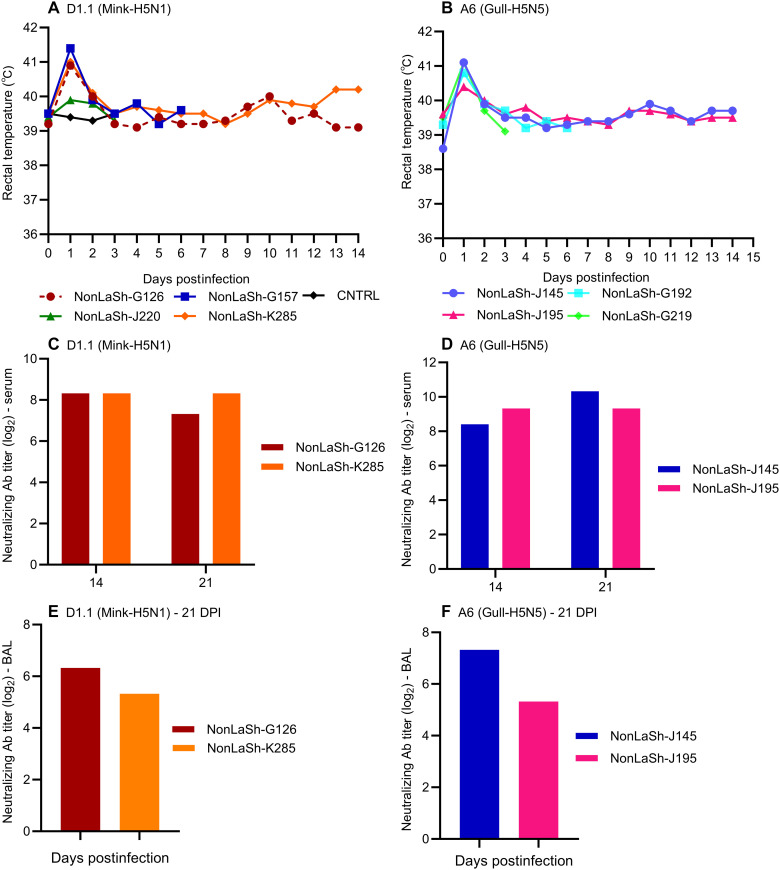
Clinical parameters and antibodies in nonlactating sheep. (**A** and **B**) Rectal temperature (°C) of infected sheep. (**C** and **D**) Neutralizing antibody (Ab) titers in serum samples. (**E** and **F**) Neutralizing antibody titers in BAL fluid. Nonlactating sheep was designated as NonLaSh. The VNT results from control noninfected sheep remained <10.

**Fig. 3.**
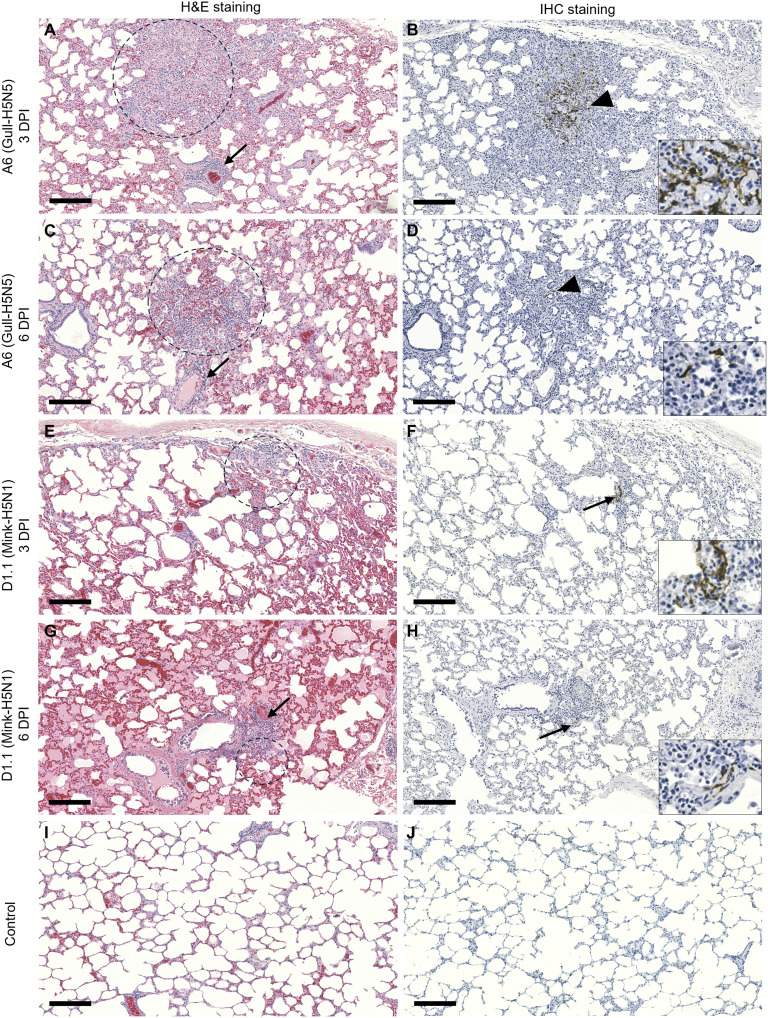
Pathology and influenza antigens in the lungs of nonlactating sheep. (**A** and **B**) Lung tissues collected from genotype A6 (Gull-H5N5)–infected sheep (NonLaSh-G192) at 3 DPI showing focal interstitial pneumonia (circle), perivasculitis (arrow), and localized areas stained for influenza NP (arrowhead). (**C** and **D**) Lung specimens collected from genotype A6–infected sheep (NonLaSh-G219) at 6 DPI showing focal interstitial pneumonia (circle), perivasculitis (arrow), and detection of influenza antigen (arrowhead). (**E** and **F**) Lung tissues collected from genotype D1.1 (Mink-H5N1)–infected sheep (NonLaSh-J220) at 3 DPI with smaller foci of interstitial pneumonia (circle), with only a few cells immunopositive for influenza NP (arrow). (**G** and **H**) Lung tissues collected at 6 DPI from genotype D1.1–infected sheep (NonLaSh-G157) showing rare peribronchial (circle) and perivascular inflammation (arrow) and only a few influenza NP antigen–stained cells (arrow) in areas of inflammation. (**I** and **J**) Lung tissue from noninfected sheep showing clear histological appearance and absence of influenza viral antigen. Insets show magnified views of the areas of immunostaining [(B), (D), (F), and (H)]. Scale bar, 200 μm.

Two groups of adult nonlactating female sheep were infected with clade 2.3.4.4b viruses through the respiratory route using a nebulizer connected to a conical mask that covered their nose and mouth (Fig. 1). They were infected with either the genotype D1.1 (A/mink/ON/FAV83/2025/H5N1; Mink-H5N1) or genotype A6 (A/GBBG/NL/OTH0021-23/2025/H5N5; Gull-H5N5) virus at a dose of 1 × 10^6^ median tissue culture infectious dose (TCID_50_) per sheep. Infected sheep showed signs of mild respiratory disease such as transient dyspnea and nasal discharge [between 2 and 4 days postinfection (DPI)] and fever reaching up to 41°C on 1 DPI and later declining to the baseline body temperature (Fig. 2, A and B). Serum samples (Fig. 2, C and D) and bronchoalveolar lavage (BAL) fluids (Fig. 2, E and F) obtained from the experimental sheep (*n* = 2 per virus) infected with either the genotype D1.1 or A6 virus, 3 weeks after infection, showed the presence of virus neutralizing antibodies. No neutralizing antibodies were detected in the serum as well as BAL fluids of a control noninfected sheep. Viral RNA was detected in the nasal swabs at multiple time points but not in the oral swabs (table S1) of some sheep. In the lungs, virus replication was observed in all lung lobes (fig. S1, A and B). At both sampling time points (3 and 6 DPI), infectious viruses were isolated from the lungs of both infection groups. Histological analysis at 3 and 6 DPI revealed mild lung lesions in both groups. These consisted primarily of a few scattered foci of interstitial pneumonia, perivascular cuffing of mononuclear cells, and peribronchitis (Fig. 3). The inflammatory foci observed in the lungs of sheep infected with the genotype A6 virus seemed prominent and diffused at both 3 and 6 DPI (Fig. 3, A and C) compared to the sheep infected with the genotype D1.1 virus that exhibited less pronounced inflammation (Fig. 3, E and G). Viral antigens were observed in cells morphologically consistent with alveolar macrophages and pneumocytes. At 3 DPI, viral antigen was diffusely detected throughout the pneumonic focus in one of the sheep infected with the genotype A6 virus (Fig. 3B). In contrast, in the A6 group at 6 DPI (Fig. 3D) and the D1.1 group at 3 DPI (Fig. 3F) and 6 DPI (Fig. 3H), only a few influenza antigen–positive cells were observed. No lesions were observed, and no antigen was detected in the control group (Fig. 3, I and J). Polymerase chain reaction (PCR) analysis of extrapulmonary tissues revealed that most samples were negative for viral RNA; however, a few contained traces near the detection limit of the PCR assay. Overall, the present study indicated that aerosolized H5Nx viruses caused mild respiratory disease consistent with the results of respiratory infection with the genotype B3.13 in infected heifers, calves, and goats (*32*–*34*). The avian- and human-type SA receptors were abundantly expressed on lung tissues, mainly on epithelial cells lining the airways (fig. S2, A and B).

### Clinical diseases and milk consistency in infected lactating sheep

**Fig. 4.**
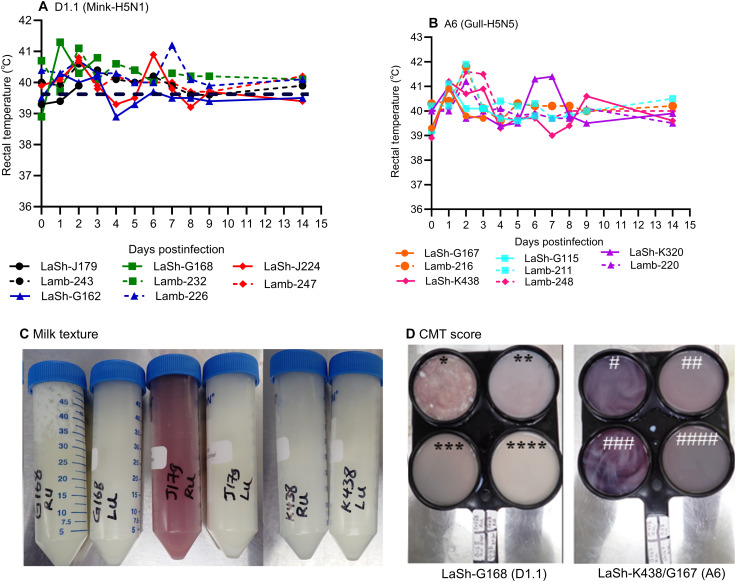
Clinical parameters, milk consistency, and CMT reactions in lactating sheep. (**A** and **B**) Rectal temperature (°C) of lactating sheep and contact suckling lambs infected with the genotype D1.1 (Mink-H5N1) or genotype A6 (Gull-H5N5) virus. Broken lines indicate the normal rectal temperature for lactating sheep and lambs. (**C**) Milk appearance from lactating sheep, LaSh-G168, LaSh-J179, and LaSh-K438 at 2 DPI (first four tubes represent milk samples collected from right and left mammary glands of sheep infected with the genotype D1.1 followed by milk samples from genotype A6–infected sheep. (**D**) CMT images/scores on milk samples: *LaSh-G168, RU-2 DPI; **LaSh-G168, left udder (LU)-2 DPI; ***LaSh-G168, RU-before infection; ****LaSh-G168, LU-before infection; #LaSh-K438, RU-2 DPI; ##LaSh-K438, LU-2 DPI; ###LaSh-G167, RU-2 DPI; ####LaSh-G167, RU-2 DPI. Lactating sheep was designated as LaSh.

This study examined the clinical effects of H5Nx infection in lactating sheep after they were inoculated with 1 × 10^5^ TCID_50_ of either the genotype D1.1 or A6 virus through the teat canal into the right mammary gland. For investigating virus transmission between mammary glands by suckling lambs, the left mammary glands were uninoculated. While no signs of respiratory disease were evident, in both groups, all lactating sheep developed fever that lasted until 3 DPI and some sheep rectal temperatures reaching 41.5°C (Fig. 4, A and B). Notably, a lactating sheep infected with D1.1 (LaSh-J224) and another with A6 (LaSh-K438) showed a recurring fever after 5 DPI, coinciding with infection of the previously uninoculated left mammary glands. Two lactating sheep (LaSh-G168 and J179) infected with the D1.1 virus developed severe necrotizing mastitis presented with swollen right mammary glands, inflamed udder skin, and ventral cellulitis extending from the udder to the brisket. Milk samples collected from the right udder (RU) of a lactating sheep, LaSh-J179 RU, contained milk with visible clots and blood compared to milk collected from the left udder (LU) (Fig. 4C). Both sheep were euthanized between 2 and 3 DPI for reaching humane end points. The other two lactating sheep in the D1.1 group, along with all four sheep infected with the genotype A6, developed mild mastitis in their RU, and milk showed slight changes in texture and consistency compared to milk derived from the left udder (Fig. 4C). California mastitis test (CMT) scores for milk samples obtained from infected right mammary glands indicated positive results as early as 1 DPI and persisted for 3 weeks (Fig. 4D and table S2), although the intensity declined over time. As expected, milk collected from uninoculated left mammary glands generally had normal consistency; in all but three sheep, two lactating sheep infected with the genotype D1.1 virus (LaSh-G168 and LaSh-J224) and another inoculated with the A6 virus (LaSh-K438) showed strong positive CMT scores, indicating virus transmission to the left mammary glands and induction of mastitis (table S2). The milk samples collected from each individual mammary gland of the lactating sheep, as well as from the uninfected control sheep, showed no CMT reactions. Given that each lactating sheep had a suckling lamb, reduction in milk volume was not quantified, but qualitative evaluation of residual milk left after suckling in the postinfection period showed a reduction when compared to preinfection yield.

### Detection of viral RNA in the swab from lactating sheep and lambs

Viral RNA was detected in the oral swabs of suckling lambs likely due to ingestion of infected milk (table S3). Infectious virus was isolated using embryonated chicken eggs from the oral swabs of three of four lambs infected with the genotype D1 and only in one of four lambs infected with the genotype A6 strain between 2 and 3 DPI. One of the lactating sheep (LaSh-K320) had detectable viral RNA in its oral swab. In contrast, nasal swab samples taken from lactating sheep and lambs showed no presence of detectable viral RNA.

### Quantification of infectious virus load and viral RNA in milk and mammary tissues

**Fig. 5.**
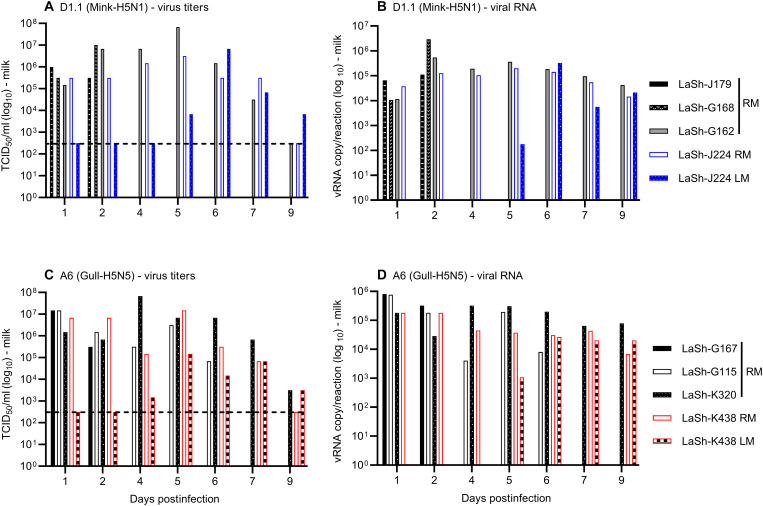
Infectious virus titers and viral RNA in milk derived from lactating sheep. (**A**) Virus titers in milk samples collected from the right and left mammary glands of lactating sheep infected with the genotype D1.1 (Mink-H5N1). (**B**) Corresponding viral RNA (vRNA) in (A). (**C**) Virus titers in milk samples obtained from the right and left mammary glands of lactating sheep infected with the genotype A6 (Gull-H5N5) virus. (**D**) Corresponding viral RNA copy in (C). When no clear CPE was detected, all wells inoculated at 10^−1^ were considered positive for CPE to generate a meaningful titer of 316 TCID_50_/ml, which was considered as the limit of detection (LOD). RT-qPCR results with Ct values >36 were considered suspicious or negative. The dashed horizontal line shows the LOD. In the figures, milk samples from the right and left mammary glands were indicated with the sheep identification number followed by RM and LM, respectively.

Milk samples collected from the right mammary glands showed higher viral titers (>1 × 10^6^ TCID_50_/ml) than the left mammary gland in both genotype D1.1–infected (Fig. 5A) and A6-infected (Fig. 5C) sheep. The shedding kinetics were comparable between both groups, with peak virus titers detected between 2 and 4 DPI. In some sheep milk, viral titers reached 6.8 × 10^7^ TCID_50_/ml. Infectious virus shedding was detected until 7 DPI, and notably, one A6-infected sheep continued to shed infectious virus until 9 DPI, although the titer (3.16 × 10^3^ TCID_50_/ml) was very low. In both infection groups, milk samples from the corresponding right mammary glands also showed strong viral RNA signals (4.2 × 10^4^ to 2.91 × 10^6^ copies per reaction), which are likely correlated with higher virus load in the milk (Fig. 5, B and D). Infection in the left mammary glands became evident as early as 4 or 5 DPI where viral titers started to peak at around 5 or 6 DPI in the milk and persisted until 9 DPI in two of the lactating sheep infected with either the genotype D1.1 [LaSh-J224 LM (left mammary)] or A6 (LaSh-438 LM) virus (Fig. 5, A and C). Viral titers in the milk samples from the left mammary gland were higher in genotype D1.1–infected sheep (up to 6.8 × 10^6^ TCID_50_/ml) relative to genotype A6–infected sheep (1.47 × 10^5^). The elevated virus titers in milk samples from the left mammary glands of sheep LaSh-J224 LM and LaSh-438 LM were substantiated by the detection of higher viral RNA in the corresponding milk samples (Fig. 5, B and D). Similarly, higher viral RNA copy was also detected in the inoculated right mammary glands of lactating sheep LaSh-G168 and LaSh-J179 infected with the genotype D1.1 (2 and 3 DPI) and in LaSh-G167 and G115 infected with the genotype A6 virus between 3 and 6 DPI (fig. S3).

### Expression of SA receptors and pathologies in mammary glands

**Fig. 6.**
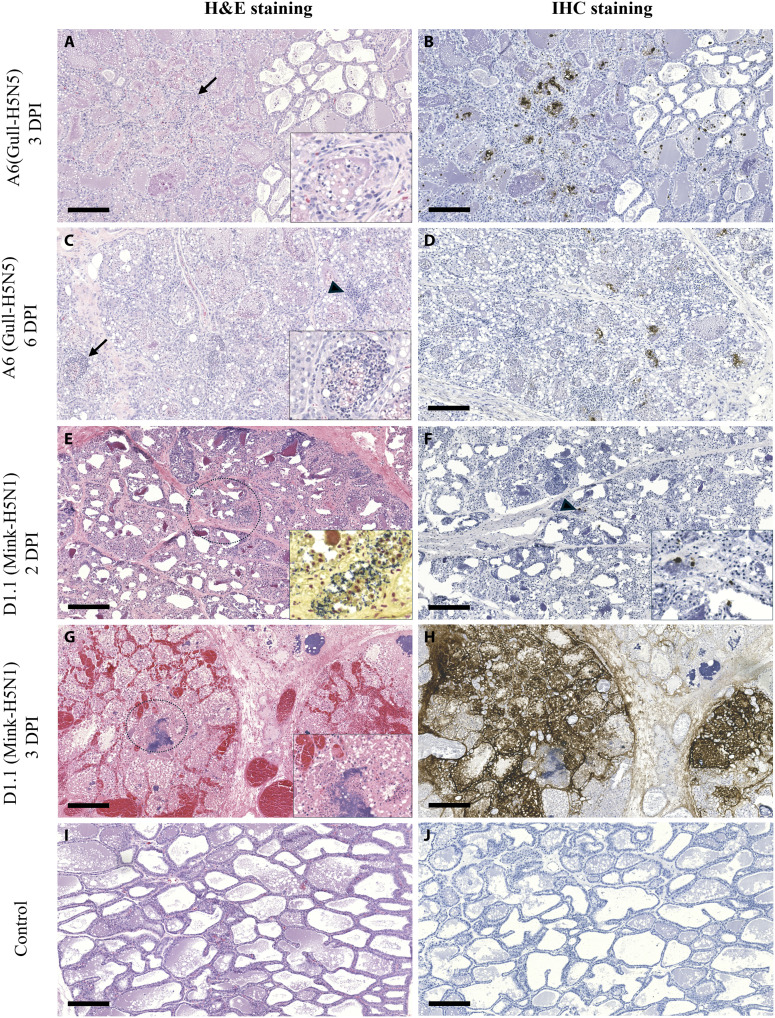
Pathology and influenza antigens in the right mammary glands of lactating sheep. (**A** and **B**) Mammary section (3 DPI) from genotype A6 (Gull-H5N5)–infected lactating sheep (LaSh-G167) showing scattered vacuolation, degeneration, and loss of alveolar epithelial cells with luminal cellular debris and occasional neutrophils (arrow, inset) and corresponding foci of immunostaining for influenza NP antigen. (**C** and **D**) Mammary section (6 DPI) from genotype A6–infected lactating sheep (LaSh-G115) showing increased foci of neutrophil infiltration (arrow, inset), foci of interstitial mononuclear inflammatory cells (arrowhead), and occasional foci of immunostaining for influenza NP antigen. (**E** and **F**) Mammary tissues collected at 2 DPI from sheep (LaSh-J179) infected with the genotype D1.1 (Mink-H5N1), revealing widespread necrosis of epithelial cells (circled) with abundant presence of bacteria (inset, Gram stain) but few foci of influenza antigen–containing cells (arrowhead, inset). (**G** and **H**) Mammary tissues collected at 3 DPI from sheep (LaSh-G168) infected with the genotype D1.1 virus, showing epithelial necrosis with bacterial infection (circle, inset) and extensive hemorrhage with diffuse staining for influenza antigen. (**I** and **J**) Mammary tissue from noninfected lactating sheep with preserved architecture of the secretory epithelial cells and no fibrosis, with no apparent viral antigen detection. Scale bars, 200 μm.

In lactating sheep, both the mammary acini and teat cisterns expressed avian- and human-type SA receptors, suggesting susceptibility to H5Nx infection (fig. S4, A to D). During the acute phase of clinical infection, in general, more pathologies and higher viral RNA or antigen were detected in the right mammary glands. The extent of pathologic alterations and the presence of viral RNA declined in the mammary glands by 21 DPI in both infection groups (fig. S3). Histologically, infected mammary glands showed necrotic secretary epithelial cells and polymorphonuclear cell infiltrations. The genotype A6 infection caused moderate mammary lesions by 3 DPI (LaSh-G167) in sheep, marked by focal areas of degeneration and necrosis (Fig. 6A). Similarly, by 6 DPI, scattered necrotic foci appeared in the right mammary gland of LaSh-G115 infected with the genotype A6, accompanied by infiltration of neutrophils and mononuclear cells (Fig. 6C). D1.1 infection caused more severe mammary lesions at both 2 DPI (LaSh-J179) and 3 DPI (LaSh-G168). Increased aggregates of inflammatory cells, extensive epithelial cell necrosis, and interlobular hemorrhages were marked in infected right mammary glands (Fig. 6, E and G). The presence of distinct bacterial colonies in the mammary tissues of these sheep suggests secondary bacterial infection, which contributes to the mammary pathology (Fig. 6E, inset). Immunohistochemistry (IHC) performed on right mammary tissues collected at 3 DPI showed few foci of viral antigen in A6-infected sheep (LaSh-G167; Fig. 6B), whereas the staining appeared diffuse and extensive in D1.1-infected sheep (LaSh-G168; Fig. 6H). Few foci of viral antigens were found in the mammary glands of an A6-infected sheep (LaSh-G115) that was euthanized at 6 DPI (Fig. 6D) and in the D1.1-infected sheep (LaSh-J179) that was euthanized at 2 DPI because of humane end points (Fig. 6F). In the control sheep, the mammary glands showed no lesions or viral antigen (Fig. 6, I and J). The right teat to which the inoculation was performed showed extensive epithelial necrosis and edema with bacterial invasion and minute amounts of viral antigen in the teat cisterns (fig. S5, A and B). Notably, the left teat cisterns from LaSh-G168 (D1.1-infected sheep) demonstrated inflammation and epithelial necrosis and abundant staining for influenza nucleoprotein (NP) antigen at 3 DPI (fig. S5, C and D), strong evidence of virus transmission to the left mammary gland. Teat cistern from control lactating sheep showed intact epithelial lining and lacked any detectable viral antigen (fig. S5, E and F). Histopathology performed on mammary glands collected at 21 DPI did not reveal any significant lesions or immunoreactivity to NP antigen in both infection groups.

### Virus detection and lesions in extramammary tissues

Efforts were made to quantify viral RNA and isolate infectious viruses from various nonmammary tissues. For reverse transcription quantitative polymerase chain reaction (RT-qPCR) analyses, cycle threshold (Ct) values ≤36 were considered significant and indicative of positive viral RNA detection. Ct values between 36 and 39 were regarded as near the limit of detection and were included only when amplification was concordant across replicates. Samples with Ct > 39 were considered negative. Using these criteria, higher viral RNA copy numbers (9.5 × 10^4^ to 1.99 × 10^5^ copies per reaction; Ct ≈ 27 to 30) were detected in the right supramammary lymph nodes (SMLs) of two lactating sheep (LaSh-G168 and LaSh-J179) infected with the genotype D1.1 virus between 2 and 3 DPI. Lower but quantifiable viral RNA (6.9 × 10^1^ to 1.83 × 10^3^ copies per reaction; Ct ≈ 33 to 36) was detected between 3 and 6 DPI in the right SMLs of two lactating sheep (LaSh-G167 and LaSh-G115) infected with the genotype A6 virus (fig. S3). The left SMLs of sheep infected with the genotype D1.1 also contained higher viral RNA loads (3.75 × 10^4^ to 3.22 × 10^5^ copies per reaction; Ct ≈ 28 to 31) compared to the A6 virus (3.95 × 10^1^ copies per reaction; Ct ≈ 36 to 37) (fig. S3). Traces of viral RNA were occasionally detected in respiratory, visceral, and reproductive tissues, particularly in lactating sheep (LaSh-G168 and LaSh-J179) infected with the genotype D1.1. Infectious virus was recovered from the liver, spleen, uterus, and ovary of these animals using embryonated chicken eggs (table S4). Histological analysis of SMLs showed lymphatic edema (fig. S6A). An extensive presence of viral antigen was detected in both marginal reticular cells (fig. S6B) and endothelial cells (fig. S6C) within the SMLs of genotype D1.1–infected sheep (LaSh-G168). While uterine lesions were less prominent (fig. S7A), both viral RNA (table S4) and antigen (fig. S7B) were detected in the uterus, indicating uterine epithelial cells as targets.

### Suckling-mediated transmission and seroconversion

Suckling lambs could acquire infection by ingesting virus-containing milk from the right mammary glands and, at the same time, transmit infection to the uninoculated left mammary glands. Within 2 to 4 days of being introduced, all suckling lambs developed fever (Fig. 4, A and B). Evidence of exposure included detection of infectious virus and viral RNA in the oral swabs of some of the lambs but at lower levels (table S3). The virus titers and viral RNA in the milk samples obtained from the left mammary glands of two of the lactating sheep after transmission were similar to the amount present in the milk from the right mammary glands (Fig. 5, A to D). This experiment underscores the “oral-to-teat” transmission route facilitated by suckling lambs. Furthermore, antibody testing detected neutralizing antibodies in the serum and milk of all lactating sheep and in the serum of lambs, confirming active infection (Table 1).

**Table 1.** Virus neutralizing antibody titers in serum and milk (whey fraction ). After an initial fivefold dilution, whey and serum were serially diluted (twofold) and incubated with either the genotype D1.1 or A6 virus. After 1 hour of incubation at 37°C, monolayers of MDCK cells were treated with the antibody-virus complex. CPEs were read between 72 and 96 hours. The neutralizing titer was calculated as the highest dilution of the samples that prevented infection of the cell monolayer. Each sample [serum, milk from the right (Rt) and left (Lt) udder] was run in duplicates. The virus neutralization test (VNT) results from control noninfected sheep remained <10.

Viruses	Sheep	DPI	Serum VNT	Viruses	Lactating sheep	DPI	Milk VNT
D1.1	LaSh-G162	14	640	D1.1	LaSh-G162-right udder	14	320
	LaSh-J224	14	320		LaSh-G162 left udder	14	320
	Lamb-226	14	80		LaSh-J224-right udder	14	320
	Lamb-232	14	640		LaSh-J224-left udder	14	160
	Lamb-243	14	640	A6	LaSh-K320-right udder	14	320
	Lamb-247	14	320		LaSh-K320-left udder	14	320
A6	LaSh-K320	14	320		LaSh-K438-right udder	14	320
	LaSh-K438	14	80		LaSh-K438-left udder	14	320
	Lamb-211	14	160	D1.1	LaSh-G162-right udder	21	640
	Lamb-216	14	320		LaSh-G162 left udder	21	640
	Lamb-220	14	320		LaSh-J224-right udder	21	1280
	Lamb-248	14	320		LaSh-J224-left udder	21	640
D1.1	LaSh-G162	21	320	A6	LaSh-K320-right udder	21	1280
	LaSh-J224	21	640		LaSh-K320-left udder	21	640
	Lamb-226	21	80		LaSh-K438-right udder	21	640
	Lamb-232	21	640		LaSh-K438-left udder	21	640
	Lamb-243	21	640				
	Lamb-247	21	320				
A6	LaSh-K320	21	1280				
	LaSh-K438	21	640				
	Lamb-211	21	640				
	Lamb-216	21	640				
	Lamb-220	21	1280				
	Lamb-248	21	640				

### Intrahost genetic diversity analysis

**Fig. 7.**
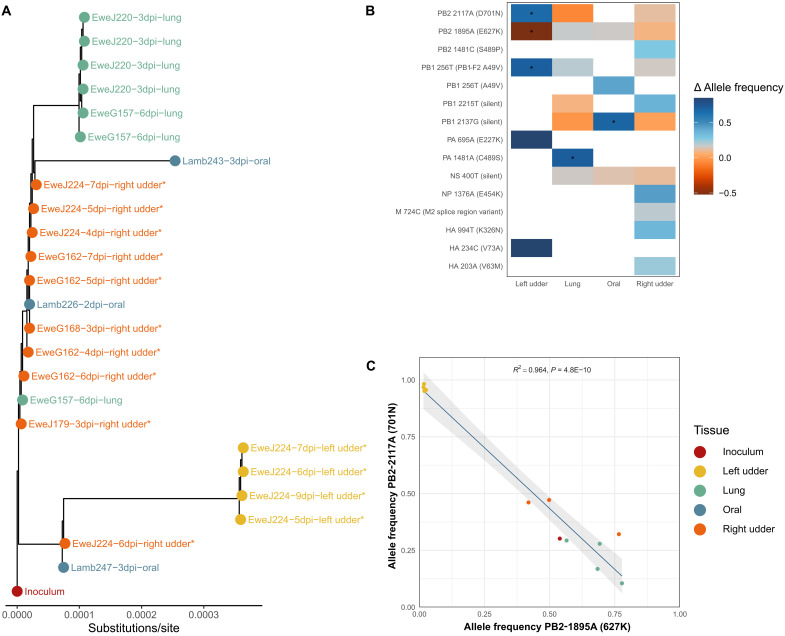
Genotype D1.1 sequences originated from tissues, swabs, and milk. (**A**) Maximum-likelihood phylogenetic tree of whole-genome D1.1 viral sequences obtained from oral swabs, milk, and tissue samples collected at various time points from both infected sheep and suckling lambs. Tree tips are colored according to the source tissue. Samples collected from lactating sheep are marked with an asterisk (*). (**B**) Heatmap of tissue-specific allele frequency changes, relative to the inoculum virus, among the whole-genome sequences presented in (A). Allelic frequency changes significantly associated with a particular tissue (*P* < 0.01), present in at least three samples, are marked with an asterisk (*). (**C**) Ordinary linear regression for allele frequencies of the mammalian adaptive substitutions PB2-E627K and PB2-D701N among whole-genome sequences presented in (A). Points are colored according to the tissue in which the sample was derived.

The Mink-H5N1 (genotype D1.1) inoculum contained a mixture of mammalian and avian adaptive residues at both PB2-627 (E/K) and PB2-701 (D/N). The phylogenetic tree based on D1.1 collected across various time points and tissues is largely segregated into tissue-specific sublineages (Fig. 7A). Viral sequences derived from milk samples collected from the right mammary glands (referred to as right udder) were the nearest to the inoculum virus (Fig. 7, A and C). The sequences derived from lung tissues (from nonlactating sheep exposed by the respiratory route) and from milk samples derived from the left mammary glands (referred to as left udder) each form their own monophyletic sublineages and showed viral variants distinct from the inoculum virus, suggestive of tissue-specific evolution (Fig. 7A). Tissue-specific changes in allele frequency postinoculum were identified in the left udder, lung, and oral samples. Left udder samples saw significant (*P* ≤ 0.01) tissue-specific increases in the frequencies of PB2-2117A [Asp^701^→Asn (D701N)] and PB1-256T [PB1-F2 Ala^49^→Val(A49V)] and a decrease in the frequency of PB2-1895A [Glu^627^→Lys(E627K)]. Lung and oral samples show increased frequencies of the alleles PA-1481A [Cys^489^→Ser(C489S)] and PB1-2137G (silent), respectively (Fig. 7B). Left milk samples saw significant increases in the frequencies of PB2-2117A (D701N) between 5 and 9 DPI (Fig. 7B). The most significant relationship between allelic frequencies was the strong negative correlation between the mutations underlying PB2-E627K and PB2-D701N (*R*^2^ = 0.964, *P* = 4.8 × 10^−10^; Fig. 7C).

### Replication of H1N1 and H5Nx in sheep mammary epithelial cells

All H5 viruses (B3.13, two D1.1 viruses, and A6) tested replicated in primary sheep mammary epithelial cells (MECs), attaining high titers within 24 hours postinfection (fig. S7). No substantial differences were observed in the replication efficiency of all clades 2.3.4.4b viruses tested. In contrast, the swine-pH1N1 virus did not replicate in cultured MECs (fig. S8).

## DISCUSSION

Two genotypes, B3.13 and D1.1, of clade 2.3.4.4b H5N1 viruses have been causing outbreaks in dairy cows across the United States (*16*, *19*, *46*). In small ruminants, H5N1 infections have been detected with the genotype B3.6 in neonatal goats (*16*) and with the European genotype DI.2 in a lactating sheep (*27*). Dairy cows (*32*, *33*) and lactating goats (*34*) experimentally infected with the genotype B3.13 by the intramammary and respiratory routes suggest that both lead to an H5N1 infection. A recent study detected infectious H5N1 virus in air samples collected from milking parlors (*47*), supporting airborne infection and spread. To confirm respiratory exposure as a potential route of entry, nonlactating sheep were challenged by aerosol means with the genotype D1.1 or A6 virus. In this study, all sheep that were exposed to the aerosolized genotype D1.1 or A6 virus exhibited a brief fever lasting 1 day, likely exacerbated by handling-related stresses. However, mild respiratory signs lingered for 3 days in most infected sheep. Plausibly, viral replication in the nasal tissues was transient, or instances of nonproductive infections resulted in transient viral RNA shedding in the nasal secretions. This could be accounted for the detection of low levels of viral RNA and, in some cases, even by the absence of detection. The aerosolized H5N1 or H5N5 virus might have reached the lower respiratory tract, leading to limited virus replication in the lungs that caused subtle pulmonary infections, resulting in milder pulmonary lesions characterized by mononuclear inflammatory cell infiltration and influenza A virus (IAV) antigen detection in inflammatory foci. These experimental findings remained consistent with the clinical outcomes observed in calves (*32*, *33*), goats (*34*), and pigs (*50*) infected with the genotype B3.13 virus. All sheep developed antibodies, with neutralizing antibodies detected in serum samples and BAL fluid, similar to what was reported from a study on aerosol-exposed heifers (*33*). This self-limiting respiratory disease and possibly low-level virus replication in the lungs and associated lymphoid tissues may trigger systemic and local antibody responses.

The genotype D1.1 virus has been circulating in North America since fall 2024 (*7*, *8*). Despite D1.1 viruses underlying sporadic outbreaks on dairy farms in the US (*46*), critical research gaps remain as there are no reports detailing the clinical impacts of D1.1 infection in dairy cows and small ruminants. At the receptor level, the mammary glands of lactating sheep express both α2,3-linked (avian-type) and α2,6-linked (human-type) SAs resembling the expression patterns in the mammary glands of lactating or pregnant cows and goats (*34*, *36*, *37*, *39*, *51*). While differences in the density and distribution patterns of the two receptors on individual epithelial cells were not assessed in colocalization imaging or other methods such as fluorescence-activated cell sorting, our findings suggest that mammary tissues in lactating mammals have the right canonical SA receptors for H5 binding and replication. Although receptor-binding assays were not performed in our study, clade 2.3.4.4b H5N1 and H5N5 viruses including genotypes B3.13, D1.1, and A6 have HA (HA1) sequences with conserved amino acid residues in the receptor-binding domain, which favor an avian-type SA binding property (*40*, *42*). In our study, genotypes D1.1, A6, and B3.13 replicated comparably in vitro using ovine primary MECs. Furthermore, to assess the pathogenicity of these viruses in an ovine model, early-lactation sheep were intramammarily inoculated with the genotype D1.1 or A6 virus into the right mammary gland, while the left mammary gland remained uninfected to monitor viral spread by suckling lambs. In general, both genotypes induced mastitis, as evidenced by positive CMT reactions. Exceptions were in two of four sheep infected with the genotype D1.1 that developed severe mastitis and produced blood-tinged milk, and histopathological analysis revealed the presence of bacteria and extensive inflammatory cell infiltrations in the mammary tissues. IHC analysis detected IAV antigens in milk alveolar spaces within all mammary tissue sections, likely in desquamated epithelial or inflammatory cells, along with the presence of extensive viral antigen staining in the entire mammary sections in D1.1-infected sheep. In this case, viral mastitis could have compromised the mammary defense mechanisms and predisposed to bacterial invasion. In a dairy herd in California, a study indicated subclinical mastitis in a significant number of dairy cows that shed elevated levels of H5N1 virus in milk (*47*). This illustrates clinical outcomes and the severity of viral mastitis to depend on a combination of factors such as the lactation stage and age of cows, host immunity, and viral genotypes (*32*, *33*, *52*). A recent study reported that dairy cows in mid- to late lactation had a higher incidence of clinical influenza compared with those in early lactation during HPAI H5N1 outbreaks on a large free-stall dairy farm in Ohio in the spring of 2024 (*31*). Experimental infections with B3.13 confirmed this trend, with older multiparous and late-lactation cows developing more severe mastitis than younger cows in early or mid-lactation (*32*, *33*). Similarly, intramammary infection with the genotype B3.13 or B1.2 in late-lactation goats triggered severe mastitis (*34*).

In days immediately after experimental inoculation, milk collected from lactating sheep contained higher infectious titers than during the later course of infection, and by 9 DPI, the infectious virus was no longer detectable. This coincided with the detection of neutralizing antibodies in the serum and milk collected from both infected and uninfected contralateral mammary glands. Similar findings were reported in dairy cows as well as lactating goats infected with the B3.13 virus (*33*, *34*). On the contrary, viral RNA was persistently detected in milk until 21 DPI, albeit at higher Ct values (>36). A similar persistence of viral RNA in milk was observed in a lactating H5 seropositive sheep in the UK (*27*) and in experimentally infected dairy cows and lactating goats (*32*–*34*, *52*). Overall, our findings suggest that mammary tropism and infection are not unique just to the B3.13 virus. The A6 (H5N5) virus, which carries a short-stalked neuraminidase (*9*, *11*), could infect mammary tissue, resulting in virus and viral RNA shedding in milk. Mammary gland histologic and immunohistochemical analyses revealed epithelial cell necrosis, polymorphonuclear cell infiltration, and widespread NP antigen detection in D1.1-infected mammary glands. In this case, bacterial co-infection appeared to exacerbate the observed lesions and virus replication. Conversely, A6 infection induced mammary pathology but with limited viral antigen distribution. Severe mammary damage appeared common following intramammary infections in goats and dairy cows with the B3.13 virus (*33*, *34*).

Beyond the mammary gland, viral RNA and antigen were detected in the SMLs, visceral organs, ovaries, and uterus. IHC suggested that lymphatic drainage from infected udder facilitated virus dissemination to SMLs, where extensive NP antigen was observed in marginal reticular cell networks within the SMLs in both lactating goats and dairy cows (*16*, *32*–*34*). While previous intramammary infection studies in dairy cows indicated the lack of systemic spread of H5N1 or H1N1 viruses (*32*, *53*), in a recent investigation in California, viral RNA was detected in longitudinally collected sera from H5N1–naturally infected dairy cows, showing that some cows with H5N1 infection develop viremia (*54*). H5N1 replication in human lung endothelial cells has been associated with viral pathogenicity and systemic viral dissemination. The presence of extensive viral antigen in endothelial cells of the SMLs in this sheep may contribute to systemic spread via the blood or lymphatic system during HPAI infections in ruminants. H5N1 infections in ovaries and uterus can affect the reproductive performance of dairy cattle and small ruminants. During the B3.13 outbreaks, reports showed cases of abortion in mid- to late-gestation heifers and disruptions of estrus cycles among dairy cows (*55*). In these cases, vertical transmission was suggested because of detection of viral RNA in lungs and brain tissues of aborted fetuses (*54*). In sea lions, during clade 2.3.4.4b outbreaks, evidence of placental necrosis in pregnant mothers as well as viral RNA detection in fetal tissues suggests vertical transmission (*56*). Instances of vertical transmission have been confirmed in pregnant and lactating mice infected with the B3.13 virus (*57*) and in a single case of pregnant woman naturally infected with H5N1 (*58*).

Viral infections can be readily transmitted through milk to newborn animals. Lactating goats infected with Powassan virus had transmitted the pathogen to their suckling offspring through milk ingestion (*59*). Similarly, domestic pigs had contracted tick-borne encephalitis virus after ingesting whey and milk from infected goats (*60*). During peak H5N1 replication in the mammary glands of dairy cows and goats, high viral loads were excreted in the milk (*32*–*34*). We suspect that lambs and goat kids with unrestricted access to suckling were exposed via oral routes as viral RNA and infectious viruses were detected in oral swabs but not in nasal swabs. Aerosolization or aspiration of milk during suckling may also lead to respiratory infections in neonates. Infected suckling lambs developed fever within days and neutralizing antibodies in the serum and BAL samples, similar to the findings seen in goat kids (*34*). Dairy calves that were bucket fed infectious milk from B3.13-infected cows showed mild clinical disease and developed higher serum neutralizing antibodies, confirming active infection (*61*). In contrast, calves that received infectious milk mixed with neutralizing antibody–rich cow milk showed partial protection against disease (*61*). Oral transmission has been confirmed in farm cats and experimentally infected mice following ingestion of raw milk from H5N1-infected dairy cows (*35*, *62*). Transmission of IAV, including H5N1 (B3.13) and H1N1, via the mammary gland-neonate dyad resulting from direct or secondary mammary infections and suckling of virus-laden milk has been demonstrated in ferrets and mice (*63*–*66*). A suspected increase in mortality among suckling elephant seal pups may be linked to the consumption of contaminated milk from lactating seals infected with the clade 2.3.4.4b H5N1 virus (*67*).

The route by which dairy cows and lactating sheep acquire H5N1 infections in their udder remains poorly understood. High viral loads in dairy milk and the ability of H5N1 to persist on milking equipment have been suggested as potential sources of cow-to-cow transmission (*48*, *68*). To investigate milking equipment–mediated infections, a recent study established the minimum intramammary infectious dose of B3.13 in dairy cows. Just 10 plaque-forming units induced mammary infection and viral shedding in milk at levels comparable to those observed following a high-dose challenge of 10^5^ plaque-forming units (*49*). However, when milking claws were shared between B3.13-infected cows and healthy cows, no viral transmission occurred. Similarly, sentinel cows cohoused with intramammarily infected animals did not develop udder infections (*49*). Longitudinal monitoring showed that the number and pattern of H5N1-positive quarters in dairy cows remained stable over time, even with shared milking claws (*47*). These findings suggest that shared milking equipment is not the primary and sole driver of H5N1 transmission between udders and cows. In naturally infected herds, it is uncommon for all four quarters of a cow to be affected, underscoring the complexity of transmission dynamics (*16*, *47*). Experimental intramammary infections with recent bovine-origin B3.13 or human-adapted H1N1 demonstrate that viral replication is largely confined to the inoculated quarters, with no spread to uninoculated glands (*33*, *52*, *53*). Compartmentalized infections likely reflect both the distinct anatomy of bovine udders and protective role of sustained antibody secretion in the milk, which may limit virus establishment in uninoculated udder (*52*). The duration of immunity after intramammary infection with B3.13 needs investigation, but an earlier report of H1N1 intramammary infections in dairy cows showed the longevity of the hemagglutination inhibition antibody (*69*).

As newborn calves are typically separated from their mothers shortly after birth, particularly in intensive dairy operations (*70*), they are unlikely to contribute to udder infections with H5N1. Following evidence of milk-borne H5N1 transmission in suckling goat kids, it was hypothesized that HPAI H5Nx viruses could be transmitted between infected and healthy mammary glands via retrograde movement along the teat canal during suckling (*34*). Here, we demonstrate the transfer of infectious virus from the inoculated right mammary gland to the uninoculated left gland of the same sheep through suckling lambs. Transmission occurred in two of four lactating sheep infected with the D1.1 strain and in one of four sheep infected with the A6 strain within 3 to 5 days. The affected left glands developed mastitis, and their milk contained high viral titers and detectable levels of viral RNA. In addition, viral antigens were identified in the epithelial cells of the left teat cistern in one of the D1.1-infected sheep. In ex vivo bovine explant cultures, the B3.13 virus replicated within the teat and gland cisterns (*71*). Supporting this mechanism, a study in lactating ferrets showed transmission of H1N1 to previously uninfected mammary glands and nipples via suckling kits (*63*). In dairy cows, a recent study demonstrated transmission of clade 2.3.4.4b H5N1 from orally infected calves but not from intranasally infected calves to the mammary glands of lactating cows despite lower virus titers in the oral swabs. The infected quarters in these cows developed severe mastitis, with viral shedding in milk, and virus replication was observed in the epithelial lining of the pharyngeal and palatine tonsils, as well as the tongue roots of infected calves (*72*). These findings from both calves and lambs suggest a potential risk of mouth-to-teat transmission of clade 2.3.4.4b H5N1 in dairy cows and lactating sheep. In addition, progressive retrograde infection via damaged teats exposed to virus-contaminated reclaimed wastewater cannot be excluded (*47*). In mixed or backyard livestock systems, suckling animals sharing water sources and pasture with domestic and wild birds during peak H5N1 seasons may facilitate transmission to the mammary glands of lactating mothers. In the British H5N1 case involving lactating sheep (*27*), grazing on contaminated pasture and self-licking around the udder similar to the conditions of self-caudal licking or cross-nursing behavior seen in grazing dairy cows plausibly facilitated viral transmission (*72*, *73*).

Low levels of H5N1/H5N5 RNA or infectious virus were detected in oral swabs from suckling lambs, likely reflecting excessive dilution by saliva or rumen juice during prolonged rumination (7 to 10 hours/day). Comparable results were observed in calves fed milk containing high H5N1 titers, which showed low viral RNA levels in saliva (*61*). In the present study, viral RNA and infectious virus, including the genotype D1.1, were recovered from some lamb oral swabs, indicating limited local replication. Receptor-binding studies in calves have shown both avian-type SA expression and human-type SA expression in oral and pharyngeal tissues (*72*), suggesting similar receptor distribution in ruminants. These findings suggest the need to optimize oral swab collection protocols for HPAI surveillance in ruminant species.

Deep sequencing of milk samples from mammary glands, oral swabs, and lung tissues revealed the emergence of viral variants distinct from the consensus sequence generated from the challenge D1.1 virus. Notably, the left mammary gland of a D1.1-infected sheep appeared to select for the variant PB2-701N, while the right gland retained mixed residues of PB2-627 (E/K) and PB2-701 (D/N). Sequence analysis suggests that the PB2-D701N mutation arose before transmission to the left gland, as oral swab from one suckling lamb already carried this mutation. Dairy cows experimentally infected with H5N1 via the intramammary route acquired PB2-E627K mutation (*32*). While D1.1 viruses from some dairy cases exhibited the mammalian adaptive mutation PB2-D701N, the genotype B3.13 from all dairy cases retained the bovine-specific PB2-M631L mutation (*74*). Both PB2-E627K and PB2-D701N provide IAVs with significant replication advantages in mammalian hosts and enhanced viral transmission (*75*, *76*).

The findings from this study have significant implications, even though there are limitations such as small sample sizes and the lack of assessment of transmission from inoculated mammary glands to uninoculated glands from the environment. However, all experimental studies in dairy cows revealed the restriction of infections and virus replication to infected quarters only. Our experimental approach has demonstrated that small ruminants are susceptible to H5N1 infections. The detection of seropositive goats and sheep during periods of heightened H5N1 activity underscores the necessity for more extensive investigations within the small ruminant herds. Once mammary infections with H5N1 have occurred in some lactating ruminants, virus can spread between the udders of lactating ruminants during suckling as some neonates could access milk from cohoused multiple lactating mothers. This scenario suggests potential for widespread virus transmission within the flocks. We suggest increased surveillance and the implementation of biosecurity measures, especially in mixed-species livestock systems or where large numbers of lactating ruminants and their neonates were cohoused or allowed to graze on communal pastures. Moreover, similar to infected dairy cows, milk obtained from infected lactating small ruminants was found to harbor higher levels of infectious viruses. This raises important zoonotic considerations in areas where raw milk consumption is common. Furthermore, the handling of infected small ruminants poses risks to human health, highlighting the need for thorough risk assessments to be carried out.

## MATERIALS AND METHODS

### Viruses

For the animal experiments, two Canadian-origin influenza viruses were included: A/mink/ON/FAV0083/2025 (H5N1) and A/great-Black-backed gull/NL/OTH0021-23/2025 (H5N5). The mink-derived H5N1 virus (Mink-H5N1) is a genotype D1.1 reassortant virus, featuring a clade 2.3.4.4b HA and a North American lineage neuraminidase (N1) gene. It has a mixture of mammalian and avian adaptive amino acid residues at PB2 positions 627 (E/K) and 701 (D/N). The gull-derived H5N5 virus (Gull-H5N5), classified as genotype A6, has circulated in Canada since 2023 (*11*, *12*). It carries a clade 2.3.4.4b HA (H5) and a Eurasian-origin N5 neuraminidase (NA) and is selected for this experiment for its virulence in conventional ferret models despite having NA stalk deletion (*11*). In addition, the genotype A6 virus (H5N5) was included to determine whether mammary infections occur with genetically distinct H5Nx viruses beyond genotype B3.13 and D1.1 H5N1 viruses reported in dairy cows. For in vitro studies in ovine-origin MECs, two additional H5N1 viruses were included: genotype B3.13 A/bovine/Texas/98638/2024 (Cow-H5N1) (*41*) and genotype D1.1 A/BC/RV04883/24 (Human-H5N1) isolated from a human case in British Columbia, Canada (*45*). A pandemic A/swine/AB/OTH33/2009 (H1N1) (*77*) served as a control for the in vitro study. All four H5 viruses were propagated in 9-day-old embryonating specific pathogen–free chicken eggs. Two days postinoculation, allantoic fluid was harvested, centrifuged, and filtered, and sterility was confirmed using standard microbiological methods (*78*). Swine-pH1N1 was propagated in Madin-Darby canine kidney (MDCK)-SIAT1 cells (*79*). Infectivity titers were calculated using the TCID_50_ on MDCK cells using the Reed and Muench method (*80*). Viral genomes from the stock virus and clinical samples were sequenced using the Oxford Nanopore Technologies’ MinION platform (SQK-RBK114.96). Virus confirmation, genomic analyses, and related methodologies were previously described (*8*).

### Source and management of sheep

Nonlactating sheep, lactating sheep, and their suckling lambs (Rideau-Dorset Crosses) were obtained from the Ontario Sheep Research Centre, Agricultural Research and Innovation Ontario, managed by the University of Guelph through its Ontario Agri-Food Innovation Alliance. The animals were transported to the National Centre for Foreign Animal Disease (NCFAD) laboratory, where they were housed in BSL-3 (biosafety level 3) animal cubicles. Their diet consisted of timothy hay cubes, alfalfa cubes, and Goalmaker 16% texturized ration (Masterfeeds, Winnipeg, MB). The sheep had ad libitum access to water. To confirm the absence of active influenza infection, nasal and oral swabs were collected and tested for IAV by real-time RT-PCR targeting the IAV matrix gene. Serum samples were also collected and screened for IAV antibodies using a competitive enzyme-linked immunosorbent assay specific to the IAV NP (*81*). Animals were acclimatized for 1 week before infection. All animal experiments were approved by the Canadian Science Centre for Human and Animal Health–Animal Care Committee under Animal Use Document (AUD#C-24-004). In addition, a risk assessment document was reviewed and approved by the Institutional Biosafety Officer before initiation of the animal experiments.

### Respiratory infection in nonlactating study

Eight nonlactating female sheep (NonLaSh) were randomly assigned into two groups (*n* = 4 per virus). Group 1 (G126, G157, J220, and K285) was infected with Mink-H5N1 (genotype D1.1), while group 2 (G192, G219, J145, and J195) was infected with Gull-H5N5 (genotype A6) (Fig. 1). The viral inoculum (1 × 10^6^ TCID_50_/ml) was aerosolized using a nebulizer device fitted with a facemask to each animal, as recently described (*34*). Animals in both groups were monitored daily for clinical signs until 21 DPI. Nasal and oral swabs were collected at 2, 3, 4, 5, 7, 10, 14, and 21 DPI to assess viral shedding. The rectal temperature was recorded daily throughout the study period. For postmortem examination, one sheep from each group was euthanized at 3 DPI (J220 from group 1 and G192 from group 2) and at 6 DPI (G157 from group 1 and G219 from group 2). The remining animals were observed until the end of the study to evaluate disease progression and recovery. Blood samples at 14 and 21 DPI and BAL samples at 21 DPI were collected for antibody testing. More than 25 different tissue samples were collected for histologic examination or RT-PCR and virus isolation. To reduce cross-contamination at sampling, only one animal was euthanized at a time. Before necropsy, the neck, brisket, and ventral abdomen including the udder of euthanized sheep were disinfected with 70% ethanol. Samples for viral RNA and virus isolation were collected while most organs were in their in situ position based on historic data on the distribution of HPAI virus in the tissues of infected dairy cattle. In the respiratory infection mode, lung and associated organs were plucked out, samples from each lobe were collected, and surgical scissors and scalpels were each time changed while sampling.

### Intramammary infection and transmission study

Nine 2-year-old sheep in early lactation (LaSh) along with nine 2-month-old suckling lambs were divided into two groups. Before infection, the lambs were separated from their mothers for 24 hours. Four lactating sheep in group 1 (G162, G168, J179, and J224) were infected via the intramammary route with Mink-H5N1 (genotype D1.1), while the other four lactating sheep in group 2 (G115, G167, K320, and K438) were infected similarly with Gull-H5N5 (genotype A6). Before inoculation, each sheep was milked to remove excess milk, and the udder was cleaned with lukewarm water. The skin and teats, including the teat meatus, were disinfected with iodine solution and 70% ethanol. The right mammary gland of each sheep was infected with 0.5 ml of virus containing 1 × 10^5^ TCID_50_ delivered via the teat meatus using a bulbed-end 20-gauge mouse oral gavage needle by insertion along the full length of the teat (Fig. 1) (*34*). The left mammary gland was not inoculated and used to assess potential virus transmission by the suckling lambs while ingesting milk from both sides of the udder. To facilitate viral entry, the inoculated teats were gently massaged in a retrograde direction to facilitate inoculum entry into the gland cisternae. No virus leakage was observed during inoculation. Following the procedure, teats and udder were disinfected to remove any surface contaminants. Twenty-four hours postinfection, the separated lambs were reunited with their respective mothers: lambs 226, 232, 243, and 247 to group 1 lactating sheep and lambs 211, 216, 220, and 248 to group 2 lactating sheep. An additional lactating sheep and lamb served as uninfected controls. Each day, animals were monitored for clinical signs, the rectal temperature was recorded, and mammary glands were inspected for swelling. Nasal and oral swabs were collected at 2, 3, 4, 5, 7, 10, 14, and 21 DPI to assess viral shedding. Milk samples were aseptically collected from each gland separately daily up to 14 DPI and at 21 DPI. Before milk collection, the udder was cleaned with lukewarm water, and the skin and teats, including the teat meatus, were disinfected with 70% ethanol. Milk from the left uninoculated glands was collected first, followed by milk from the right inoculated glands, and organoleptic properties were assessed. As each lamb suckled from its dam, residual milk after suckling was collected, and the volume was compared with preinfection levels. Fresh milk collected from the right and left mammary glands was tested for indicators of mastitis using the California Mastitis Test kit (Jorgensen Laboratories, CO) pre- and postinfection. In addition, milk samples collected from the control uninfected sheep were assessed for evidence of mastitis. The CMT reactions were recorded and scored as described recently (*34*). Because of severe necrotizing mastitis, two lactating sheep from the Mink-H5N1 (D1.1)–infected group (J179 and G168) were euthanized at 2 and 3 DPI for humane end points. In the Gull-H5N5 (A6) virus group, sheep G167 and G115 were euthanized at 3 and 6 DPI. The control sheep and its lamb were euthanized at 3 DPI. The remaining lactating sheep and all lambs were maintained until the end of the study. Blood samples for antibody testing were collected from lactating sheep and lambs at 14 and 21 DPI. Tissue collection and processing procedures were consistent with what have been described for the respiratory infection experiment. The mammary glands and associated lymph nodes were collected at the end of each necropsy.

### Isolation and infection of sheep primary MECs

Detailed protocols for the isolation and culture of MECs were described in our recent article (*34*). MECs were seeded at a density of 2 × 10^5^ cells per well onto 12-well Transwell inserts (0.4-μm pore size, transparent PET membrane; Corning) precoated with human type IV collagen and bovine fibronectin. Cells were cultured under submerged growth conditions for 1 week where the apical and basolateral compartments received 500 and 1200 μl of media. Before infection, cells were washed with plain Dulbecco’s Modified Eagle Medium (DMEM). The apical surface was then infected at a multiplicity of infection of 0.1 with one of the following viruses: Mink-H5N1 (D1.1), Human-H5N1 (D1.1), Cow-H5N1 (B3.13), Gull-H5N5 (A6), or Swine-pH1N1. All infections were performed in triplicate in two independent experiments. After 2 hours of infection, MECs were washed three times on both the apical and basolateral surfaces with plain DMEM. Fresh DMEM/F-12 medium supplemented with 1% bovine serum albumin, 1% penicillin/streptomycin (P/S), 1% l-glutamine, and 1% MEM Non-Essential Amino Acids Solution was added to both compartments. The cells were incubated at 37°C with 5% CO_2_, and the apical supernatant was collected at 8, 24, 48, and 72 hours postinfection to assess viral replication kinetics in MDCK cells. All centrifugation was performed at 450*g* for 5 min.

### Detection of viral RNA in swabs, milk, and tissues

Milk samples were diluted 1:3 (v/v) in PrimeStore Molecular Transport Medium (Longhorn Vaccines and Diagnostics LLC of Bethesda, MD). Tissue samples were homogenized in 1× phosphate-buffered saline using a Precellys 24 Touch Homogenizer (Bertin Technologies) for two cycles of 20 s each, generating 10% (w/v) homogenates, which were immediately placed on ice. Homogenates were centrifuged at 3000*g* for 20 min at 4°C, and the supernatant was collected. RNA was extracted from swabs, milk, and tissue homogenates using the MagMax 1835 Nucleic Acid Isolation Kit (Thermo Fisher Scientific). During extraction, enteroviral armored RNA (ARM-ENTERO; Asuragen) was spiked into each sample as an exogenous extraction and internal reaction control. Detection of IAV RNA was performed by targeting the matrix gene (M1) using real-time RT-qPCR (*77*). A standard curve was generated from a 10-fold dilution series (in triplicates) of an in vitro–synthesized RNA template targeting the matrix gene (M1) of IAV. The matrix gene–specific RNA copy numbers in the samples (milk, tissue homogenates, and swabs) were calculated on the basis of the linear regression of the standard curve in the LightCycler480 System (Roche Diagnostics), and the efficiency was calculated at 1.971 and an error of 0.0126. The results were expressed as log_10_ viral RNA copy numbers per reaction. All apparent controls (no-template control and positive controls) were included while running each PCR run. Validated RT-qPCR results for M1 gene with Ct values ≤36 were considered positive, and >36 was considered suspicious or reported as a negative result. This threshold was established by benchmarking this assay with protocols used by international reference laboratories.

### Virus titration in MDCK cells and isolation in chicken eggs

Infectious virus load in the milk samples and infected MECs and isolation of infectious viruses from tissue homogenates and swab samples with relatively high viral RNA copy numbers were described recently (*34*). Virus titers were calculated using the Reed and Muench method (*80*). Allantoic fluid harvested from dead or chilled eggs was subjected to a hemagglutination test to evaluate the presence of replicating viruses (*78*).

### Histological examination, IHC, and SA staining

Formalin-fixed tissue samples were trimmed, embedded in paraffin, and processed according to standard procedures. Tissue sections (5 μm thick) were stained with hematoxylin and eosin (H&E) and evaluated by an American College of Veterinary Pathologists board–certified veterinary pathologist. For IHC analysis, tissues from lungs, mammary glands, SMLs, and uterus with strong Ct values in RT-qPCR (Ct values <33) were stained for IAV NP, as previously described with all standard controls (*81*, *82*). The NP monoclonal antibodies used for IHC staining of IAV antigen were validated for specificity in several temporal experimental infections with H5N1 in specific pathogen–free chickens and goats, and patterns and extents of IAV antigens in IHC-stained tissue sections were compared with in situ hybridization–stained sections (*34*, *81*). The tissue sections were then counterstained with Gill’s 1 hematoxylin to assess the extent and distribution of IAV antigen. To detect avian- and human-type SA receptors (SAα2,3-Gal and SAα2,6-Gal), tissue sections from mammary glands, teat cisterns, and lungs were stained with biotinylated *Maackia amurensis* lectin II (MALII; B-1265-1) and *Sambucus nigra* lectin (SNA; B-1305-2), respectively. Streptavidin-conjugated DyLight 488 (Vector Laboratories) was used for signal detections. Images were acquired using either an Olympus confocal microscope or PhenoCycler-Fusion system. The specificity of these antibodies was described in our recent study (*34*).

### Antibody testing

Virus neutralizing antibodies in serum, BAL fluids, and milk samples were measured as described previously (*83*), with modification in sample preparation. MDCK cells were seeded at 1.5 × 10^4^ cells per well into a 96-well tissue culture plate (Corning) in DMEM (Multicellular) supplemented with 10% heat-inactivated fetal bovine serum and 1% P/S and incubated overnight at 37°C in 5% CO_2_ to reach about 85% confluency. BAL fluid (50 ml) was obtained by adding sterile 1× phosphate-buffered saline into the trachea and lungs of euthanized sheep. The BAL fluid was promptly vortexed, and the mucous layer along with cell debris was removed through centrifugation at 3000 rpm for 20 min at 4°C. The upper mucous layer was carefully discarded, and the aqueous phase was collected without disturbing the cellular pellet, aliquoted, and frozen for later analysis. Milk samples were treated with rennet (150 μg per 1 ml of milk; Millipore Sigma catalog no. R5876-50G) for 10 min at 37°C until curdling occurred. The samples were centrifuged at 1500*g* for 10 min to separate the whey fraction, which was then heated at 63°C for 30 min. Serum and BAL fluids were inactivated at 56°C for 30 min. All samples (sera, BAL, and whey fraction) were then diluted 1:4 in 96-well U-bottom plates, followed by twofold serial dilutions in DMEM. Each dilution was then mixed with 100 TCID_50_ per well of clade 2.3.4.4b A/H5Nx viruses (Mink-H5N1 or Gull-H5N5) and incubated at 37°C for 1 hour. The antibody-virus complex was added to preformed MDCK cell monolayers in DMEM containing 1% bovine serum albumin and 1% P/S and incubated at 37°C in a 5% CO_2_ incubator. Cells were monitored daily for cytopathic effects (CPEs) up to 5 days postinfection. All samples were tested in duplicates. The highest dilution that resulted in 100% protection of the cell monolayer from CPEs was recorded as the neutralizing antibody titers. Neutralization titers below the detection limits (<10) were recorded as 10. The results from both control and infected sheep were tabulated.

### Genome sequencing and SNP analysis

To assess viral evolution, we performed whole-genome sequencing on viral RNA obtained from oral swab, milk, and tissue samples collected at various time points from both infected sheep and suckling lambs. Swab, milk, and tissue samples were subject to sequence library preparation with the Oxford Nanopore Rapid Barcoding Kit (SQK-RBK110.96 or SQK-RBK114.96), sequenced with MinION R9.4.1 or R10.4.1 Flow Cells (FLO-MIN106D or FLO-MIN114) on an Oxford Nanopore GridION sequencer (Oxford Nanopore Technologies), and processed using nf-flu (versions 3.3.5 to 3.10.1; https://zenodo.org/records/17127157). Individual viral segments from each sample were trimmed of regions flanking the open reading frames and concatenated into a whole-genome sequence (in the order of PB2, PB1, PA, HA, NP, NA, M, and NS). Concatenated genomes were aligned using MAFFT version 7.49 and used to estimate a maximum likelihood phylogenetic tree with IQ-TREE version 2.2.0 under the best-fitting model of nucleotide substitution, as determined by ModelFinder. Node support for the resulting tree was assessed by 5000 ultrafast bootstrap replicates and rooted on the inoculum virus. Allele frequencies for all variable nucleotide positions were extracted from variant reports generated by nf-flu and compared to inoculum viruses. Variable nucleotide positions with sequence coverage <10 reads were ignored. To assess whether tissue type was associated with the emergence of specific allelic frequency changes postinoculum, a linear model of allele frequency as a function of tissue was fit, and estimated marginal means were computed for each tissue-allele pair (present in at least three samples) using the emmeans R package (version 2.0.1). Tissue-specific allele frequency changes (relative to the inoculum) were assessed for significance using *t* tests of tissue-versus-inoculum contrasts. False discovery rate correction for multiple comparisons was performed by the Benjamini-Hochberg method using the p.adjust() function in the stats R package (version 3.6.2). As the frequencies of mammalian adaptive substitutions PB2-E627K and PB2-D701N varied greatly by tissue type, we assessed whether any other alleles were coevolving with these two substitutions. For each sample, the predictor alleles (PB2-627K or PB2-701N) were recursively paired with all other variable nucleotide positions. For each unique pair of alleles, present in at least three samples, an ordinary linear regression was fit to their allelic frequencies using the lm function in base R. For each regression, slope statistics (standard error, *R*^2^, and *P* value) were extracted using the broom R package (version 1.0.11) and multiple testing correction performed as above.

### Statistical analysis

All statistical analyses were conducted using GraphPad Prism (version 10, GraphPad, La Jolla, CA). Graphs were generated also using Prism software. Differences in virus replication kinetics in primary MECs among the five viruses were analyzed by a one-way analysis of variance (ANOVA). Comparisons were made between viruses at individual time points. Data were presented as the means ± standard error of the mean (SEM). *P* < 0.05 was considered statistically significant.
