# Supplementary material for: Mammary and respiratory infection of sheep with H5Nx clade 2.3.4.4b viruses with milk-mediated transmission to lambs
Source: Sci Adv. 2026 May 8;12(19):eaed1287. doi: 10.1126/sciadv.aed1287 (PMC13155352; doi:10.1126/sciadv.aed1287)
Supplement: Supplementary file 1 — Figs. S1 to S8 Tables S1 to S4 [file sciadv.aed1287_sm.pdf]

Science Advances
AAAS

Supplementary Materials for

Mammary and respiratory infection of sheep with H5Nx clade 2.3.4.4b viruses with milk-mediated transmission to lambs

Tamiru N. Alkie *et al.*

Corresponding author: Yohannes Berhane, yohannes.berhane@inspection.gc.ca

Sci. Adv. **12**, eaed1287 (2026)
DOI: 10.1126/sciadv.aed1287

This PDF file includes:

Figs. S1 to S8
Tables S1 to S4

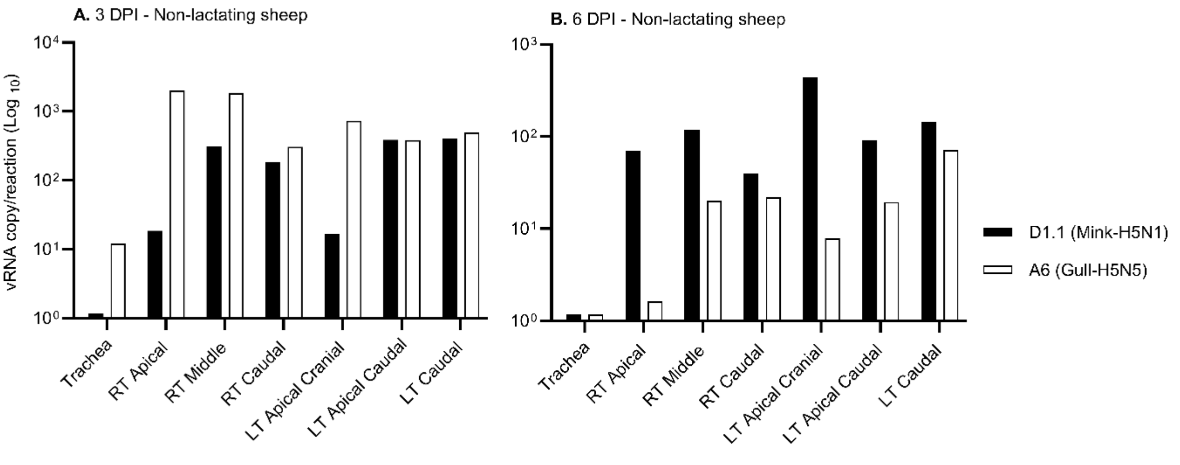

Fig. S1.

Viral RNA in trachea and lung lobes of non-lactating sheep. Ten percent tissue homogenates were prepared in 1x PBS, and viral RNA was detected using RT-qPCR assay and quantified based on standard curve generated from *in vitro*-synthesized RNA from IAV matrix gene (M1). (**A**) Virus RNA at 3 DPI, (**B**) Virus RNA at 6 DPI (genotype D1.1 or A6 virus infection). Samples with Ct-values >36 was reported as suspicious or negative.

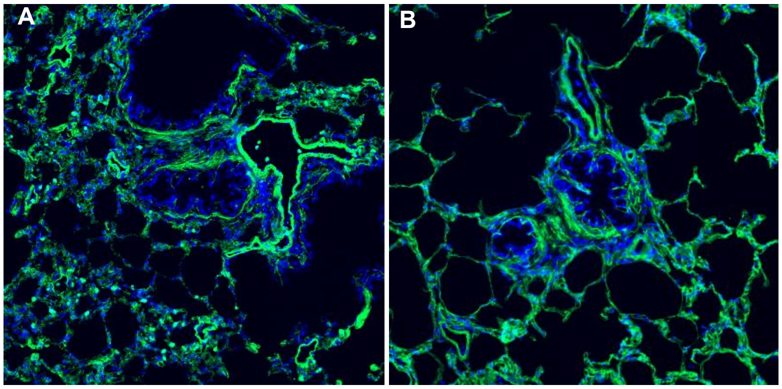

Fig. S2.

Expression of sialic acid receptors in the lungs of control (uninfected) sheep. (A) Human type sialic acid receptor (SAα2,6-Gal), (**B**) Avian type sialic acid receptor (SAα2,3-Gal) in the lungs.

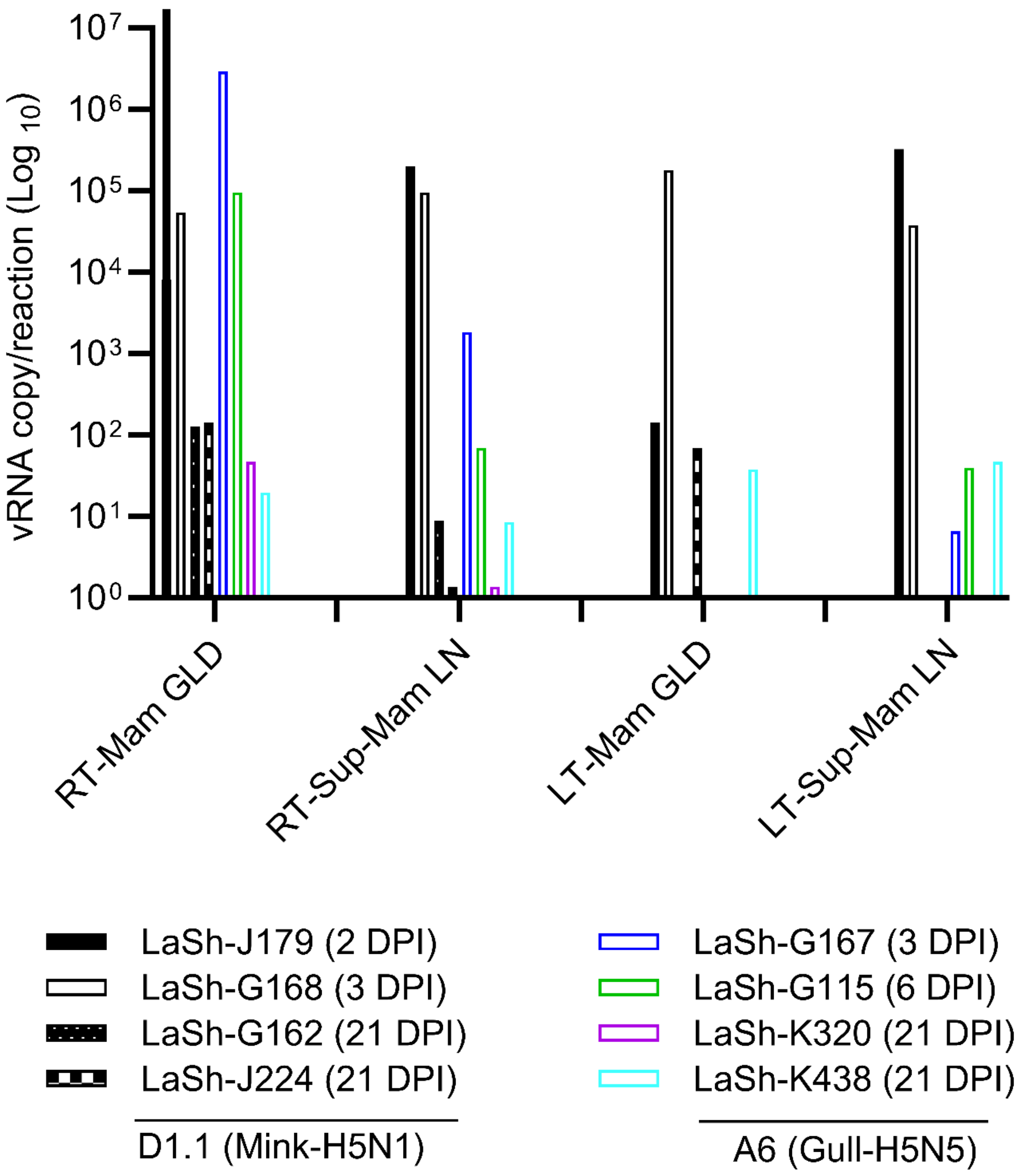

Fig. S3.

Viral RNA in mammary glands and supramammary lymph nodes of lactating sheep. Ten percent homogenates were prepared from the right (RT) and Left (LT)-mammary glands, and the corresponding supramammary lymph nodes and viral RNA were detected using RT-qPCR assay and quantified based on standard curves generated from *in vitro*-synthesized RNA from IAV matrix gene (M1). Samples with Ct-values >36 was reported as suspicious or negative.

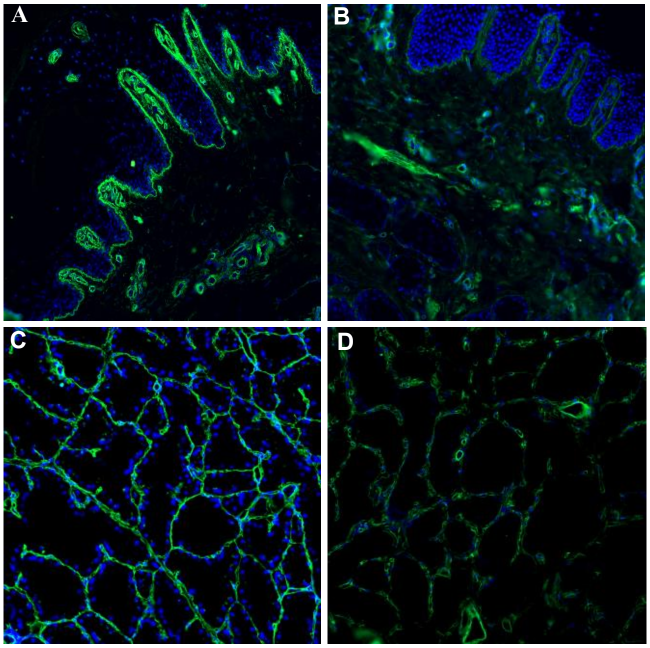

Fig. S4.

Expression of sialic acid receptors in the mammary tissue of control sheep. **(A)** Human type sialic acid receptor and (**B**), Avian type sialic acid receptor in the teat cisterns, (**C**) Human type sialic acid receptor and (**D**) Avian type sialic acid receptor in the mammary glands of a lactating sheep.

.

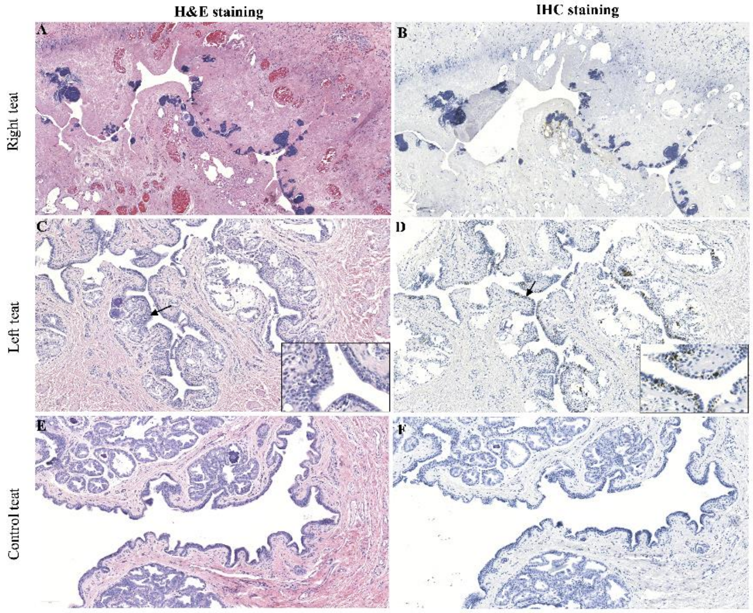

Fig.S5.
Pathology and influenza antigen detection in teat cisterns. (A) Right teat histology showing extensive epithelial cell necrosis, edema with bacterial invasion, (**B**) IHC stained right teat section with traces of virus antigen, (**C**) Left teat histology showing areas of epithelial cell necrosis (arrow and inset), (**D**) IAV antigen detected in affected left teat cistern, mainly in epithelial cells (arrow and inset). (**E**) Teat cistern from control lactating sheep, (**F**) No immuno-stained for IAV NP. The right and left teat were collected from genotype D1.1 infected LaSh-G168 at 3 DPI**.**

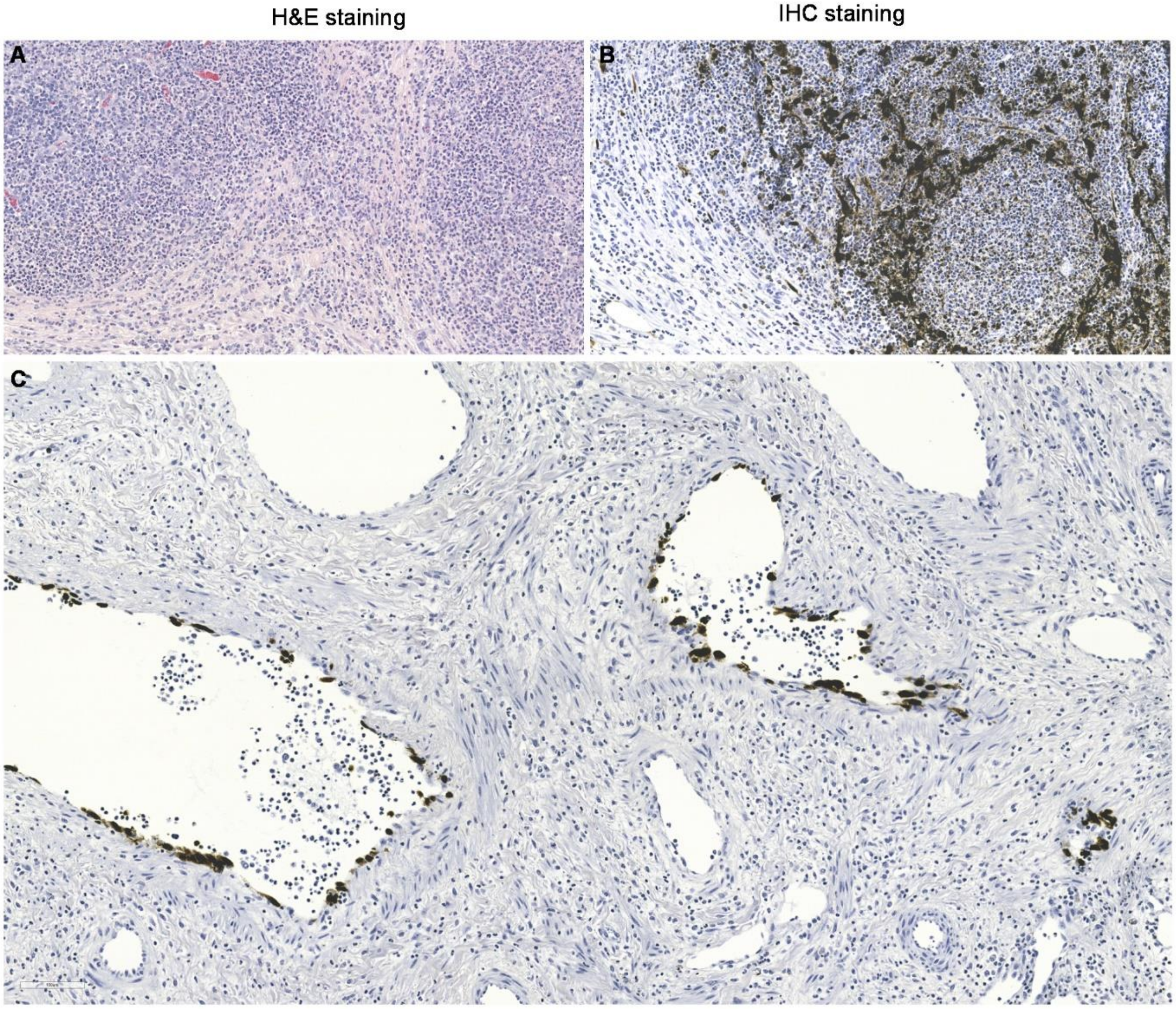

Fig.S6.

Histologic and immunohistochemical analysis of supramammary lymph node (SML). (**A**) Mild edema, **(B)** Extensive influenza antigen detected in marginal reticular or macrophages, (**C**) IAV antigen-stained endothelial cells within SML.

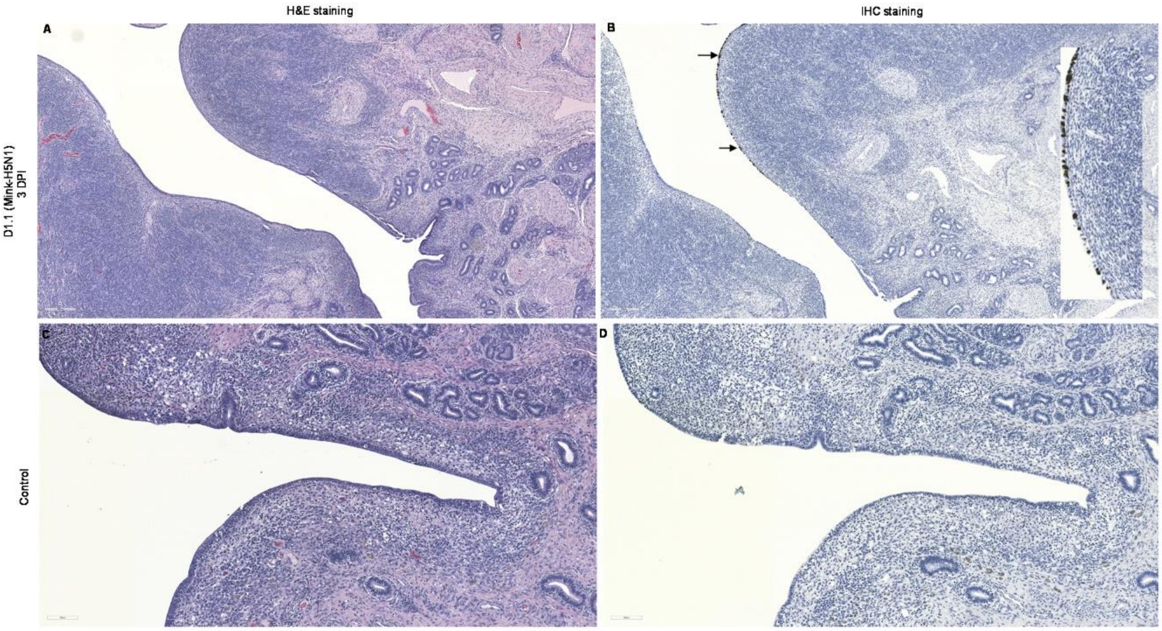

Fig.S7.

Pathology and influenza antigen detection in uterus. (A) H & E-stained uterine section, intact epithelial lining, (**B**) Foci of uterine epithelial cells immunostained for IAV nucleoprotein (arrows and inset), (**C**) Uterine section obtained from control sheep, (**D**) IHC staining for IAV nucleoprotein in the uterus of control sheep, no antigen detected. The uterine tissues were collected from genotype D1.1 infected LaSh-G168 at 3 DPI**.**

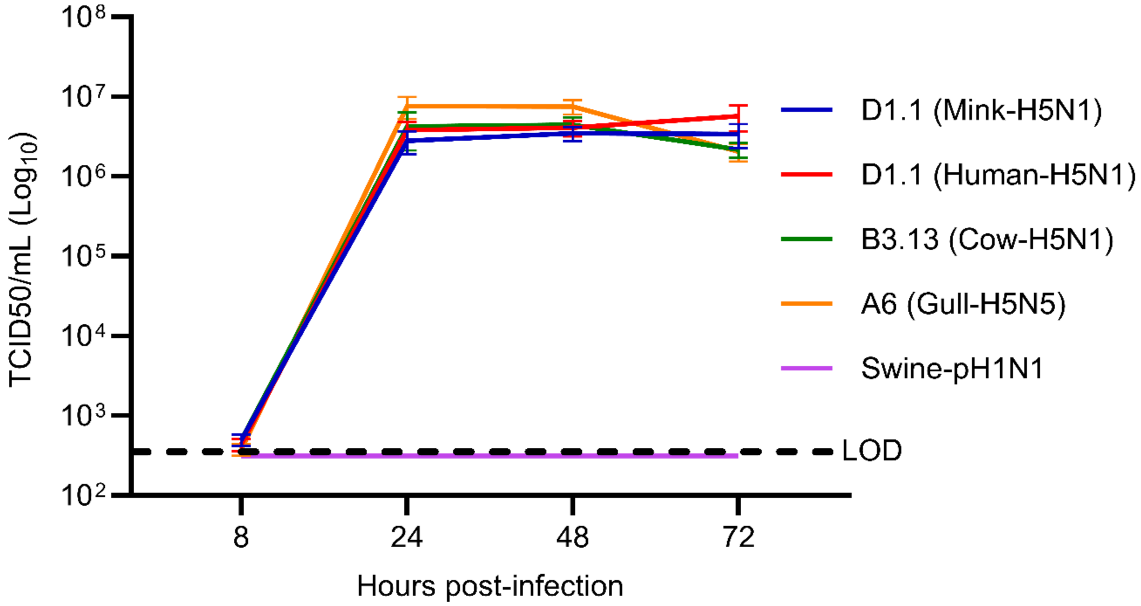

Fig. S8.
Replication kinetics of H5Nx and H1N1 in ovine mammary epithelial cells. Primary mammary epithelial cells (MECs) were obtained from a lactating sheep, cultured on porous transwell insertse system in a submerged state. MECs were infected with H5Nx or H1N1 from the apical surfaces at a multiplicity of infection (MOI) of 0.1 for 2 hrs. The inoculum was removed, and MECS were washed thrice, and they were cultured in DMEM/F-12 medium in a submerged condition. The cells were incubated at 37°C with 5% CO_2, and apical supernatant was collected between 8- and 72-hours post-infection to assess viral replication kinetics in MDCK cells and expressed as TCID50/mL. Each experiment had n = 3-4 biological replicates and performed in two independent trials. Data were presented as the mean ± standard error of the mean (SEM). A p-value of < 0.05 was considered statistically significant.

Viruses	Non-Lactating sheep	Nasal swabs			
		2 DPI	3 DPI	4 DPI	5 DPI
D1.1 (Mink-H5N1)	NonLaSh-J157	0.96	78	23.9	0.96
	NonLaSh-G126	0.96	0.96	0.96	7.29
	NonLaSh-J220	2.78	3.23		0.96
	NonLaSh-K285	2.05	2.84	6.59	0.96
A6 (Gull-H5N5)	NonLaSh-J145	0.96	0.96	0.96	0.96
	NonLaSh-J192	178	560	802	5190
	NonLaSh-G195	0.96	.96	0.96	1390
	NonLaSh-G219	408	691		0.96
		Oral Swabs			
D1.1 (Mink-H5N1)	NonLaSh-J157	0.96	0.96	0.96	
	NonLaSh-G126	0.96	0.96	0.96	0.96
	NonLaSh-J220	0.96	14.2		0.96
	NonLaSh-K285	2.92	0.96	0.96	0.96
A6 (Gull-H5N5)	NonLaSh-J145	0.96	0.96	0.96	1.76
	NonLaSh-J192	2.84	118	5.96	17.3
	NonLaSh-G195	0.96	0.96	1.4	0.96
	NonLaSh-G219	0.96	0.96		0.96

Table S1.

IAV RNA detected in the nasal and oral swabs of non-lactating sheep.

RT-qPCR results with Ct-value >36 were considered suspicious or negative results. Data were reported as viral RNA copy numbers, whenever there were traces still detectable.

**Virus**	Sheep	CMT score (Right/Left udder) at days post-infection (DPI)									Milk appearance
		1	2	3	5	6	7	9	14	21	
D1.1	LaSh-J179	$2^{+}/-$	$3^{+}/1^{+}$								Blood tinged
	LaSh-G168	$2^{+}/1^{+}$	$3^{+}/1^{+}$	$3^{+}/2^{+}$							Thicker with slight clots and blood-tinged
	LaSh-G162	$1^{+}/-$	$2^{+}/-$	$2^{+}/-$	$2^{+}/-$	$2^{+}/-$	$2^{+}/-$	$2^{+}/-$	$1^{+}/-$	-/-	No significant alterations, fewer clots
	LaSh-J224	$1^{+}/-$	$2^{+}/-$	$2^{+}/-$	$2^{+}/1^{+}$	$2^{+}/2^{+}$	$2^{+}/2^{+}$	$2^{+}/2^{+}$	$1^{+}/1^{+}$	$1^{+}/1^{+}$	Slightly thicker milk
A6	LaSh-G167	$1^{+}/-$	$2^{+}/-$	$2^{+}/-$							No significant alterations, fewer clots
	LaSh-G115	$2^{+}/-$	$2^{+}/1^{+}$	$2^{+}/-$	$2^{+}/-$	$2^{+}/-$					Slightly viscous/thicker milk
	LaSh-K320	$1^{+}/-$	$2^{+}/-$	$2^{+}/-$	$2^{+}/-$	$2^{+}/-$	$2^{+}/-$	$2^{+}/-$	$1^{+}/-$	-/-	Scant clots
	LaSh-K438	$1^{+}/-$	$2^{+}/-$	$2^{+}/1^{+}$	$2^{+}/2^{+}$	$2^{+}/2^{+}$	$2^{+}/2^{+}$	$2^{+}/2^{+}$	$1^{+}/1^{+}$	$1^{+}/1^{+}$	Slightly thicker milk

Table S2.

CMT scores and milk texture in infected lactating sheep. Two groups of lactating sheep (n = 4/virus) were intramammarily inoculated with 10^5 $TCID_{50}$ of genotype D1.1 or A6 virus (500 µL/teat) via the teat canal into the right mammary gland. The left mammary gland was uninoculated to assess virus transmission by suckling lambs that were reunited with their mothers on 1 DPI. Milk samples collected from the left and right mammary glands were inspected for changes in texture and consistency. Milk samples collected from each individual mammary gland of the lactating sheep prior to infection, as well as those from the uninfected control sheep, did not exhibit any CMT reactions. The CMT reactions were graded as + = 1^{+}; ++ = 2^{+} and +++ = 3^{+}.

Viruses	Sheep	Oral Swabs			
		2 DPI	3 DPI	4 DPI	7DPI
D1.1 (Mink-H5N1	LaSh-J179	UD			
	LaSh-G168	UD			
	LaSh-G162	UD	UD	UD	UD
	LaSh-J224	UD	UD	UD	UD
	Lamb-226	406	18.3	2.44	UD
	Lamb-232	141	UD	UD	UD
	Lamb-243	13	405	UD	UD
	Lamb-247	11.7	162	193	141
A6 (Gull-H5N5)	LaSh-G167	UD	UD		
	LaSh-G115	58.7	UD	UD	
	LaSh-K320	UD	UD	901	UD
	LaSh-K438	UD	UD	UD	UD
	Lamb-211	80.2	UD	UD	UD
	Lamb-216	5.02	UD	3.23	UD
	Lamb-220	41.0	UD	UD	UD
	Lamb-248	UD	UD	UD	UD

Table S3.

IAV RNA in oral swabs of lactating sheep and suckling lambs. Nasal swabs were negative. RT-qPCR results with Ct-values >36 were considered suspicious or negative result. UD = undetermined. Data were reported as viral RNA copy numbers, whenever there were traces still detectable.

Tissues	D1.1 (Mink-H5N1)		A6 (Gull-H5N5)
	LaSh-J179<br>2 dpi	LaSh-G168<br>3 dpi	LaSh-G115<br>6 dpi
Nasal turbinate			
Retropharyngeal LN	80.9*	38.2	1.37
Tracheobronchial LN		1.47	
Trachea	88.4	1.47	
Right apical lobe lung	147*		
Right middle lobe lung	7.04	1.47	
Right caudal lobe lung	7.62		2.24
Left apical cranial lobe lung	5.4		
Left apical caudal lobe lung	1.47		
Left caudal lobe lung	1.47		
Heart			
Liver	53.2*	50.9*	
Spleen	100*	187*	
Kidney		17.7	
Adrenal gland	1.47		1.37
Pancreas		73.5	
Duodenum			
Jejunum	1.47		2.26
Ileum	1.47	19.1	1.37
Proximal colon		1.47	2.44
Cecum		1.47	
Uterus	67.1*	21300*	1.37
Ovary	193*	1.47	
Cerebrum			
Cerebellum			
Brainstem			

Table S4.
Detections of IAV RNA in extramammary tissues of lactating sheep.
Tissues with strong Ct-values were inoculated into embryonated chicken eggs as described in the materials and methods. Embryonic mortality was recorded and allantoic fluid collected, and hemagglutination activity was determined and presence of IAV RNA was confirmed in RT-qPCR. Samples with Ct-values (>36) for influenza matrix gene (M1) were concluded as suspicious or negative. Data were reported as viral RNA copy numbers, whenever there were traces still detectable. No viral RNA was detected in extramammary tissues of lactating sheep (LaSh-G167) infected with genotype A6 virus. Asterisks (*) indicate successful infectious virus isolation in embryonated chicken eggs.
